# Predictive value of osteopenia as prognostic marker for survival and recurrence in patients with gastrointestinal cancers: a systematic review and meta-analysis

**DOI:** 10.3389/fmed.2025.1527829

**Published:** 2025-05-01

**Authors:** Xinmei Zou, Yang Wang

**Affiliations:** Ward 13 (Respiratory Digestive Geriatrics), Huzhou Third Municipal Hospital, The Affiliated Hospital of Huzhou University, Huzhou, Zhejiang, China

**Keywords:** osteopenia, GI cancers, overall survival, recurrence-free survival, osteosarcopenia

## Abstract

**Background:**

Early detection, systematic prevention, and personalized therapy are crucial to reduce mortality in patients with gastrointestinal (GI) cancers. This systematic review and meta-analysis aimed to clarify the predictive value of osteopenia and osteosarcopenia as prognostic markers of survival and recurrence in patients with GI cancers.

**Methods:**

Medline, Google Scholar, and Science Direct databases were searched for English-language studies that included patients who underwent surgical resection following a pathologically diagnosed GI cancer and reported the association between osteopenia and osteosarcopenia on the overall survival (OS) and recurrence-free survival (RFS). Meta-analysis was done using STATA 14.2, and the results were reported as pooled hazard ratios (HR) with 95% confidence intervals (CI). Heterogeneity was assessed using the I2 statistic and the Chi-square test. Study quality was evaluated using the Newcastle Ottawa Scale (NOS).

**Results:**

A comprehensive literature search yielded 23 eligible studies, primarily from Japan. Osteopenia emerged as a significant risk factor for both OS (pooled HR 2.20, 95% CI: 1.74–2.79) and RFS (pooled HR 2.15, 95% CI: 1.60–2.89). Patients with osteosarcopenia exhibited threefold higher mortality rates (pooled HR 2.96, 95% CI: 1.99–4.40) and heightened risk of recurrence (pooled HR 2.75, 95% CI: 1.79–4.24). Subgroup analyses underscored the consistency of these associations across diverse contexts.

**Conclusion:**

This meta-analysis establishes osteopenia and osteosarcopenia as robust prognostic indicators for survival and recurrence in GI cancers. Integrating musculoskeletal assessments into routine oncological care is imperative for timely interventions and optimized patient outcomes.

## Introduction

Gastrointestinal (GI) cancers include malignancies of the esophagus, stomach, pancreas and biliary apparatus, liver and colon (1). GI cancers represent a formidable global health challenge, contributing significantly to morbidity and mortality (2). The prognosis of GI cancers may be influenced by many factors, such as tumor size, extent of metastases, and musculoskeletal status of patients that emerges as a critical determinant of overall well-being (3, 4).

Numerous studies have focused on the relationship between body composition and cancer prognosis. Recent reports have shown that osteopenia, characterized by low bone mineral density [BMD], sarcopenia, marked by loss of skeletal muscle mass, and osteosarcopenia, defined as the coexistence of osteopenia along with sarcopenia in cancer patients, are conditions that may potentially impact GI cancer outcomes (5, 6). Low BMD is often linked with an increased risk of falls, fractures, hospitalization, and even death, thereby negatively impacting the health-related quality of life (5). Additionally, bone loss in cancer patients may reflect osteopenia, malnutrition, and systemic inflammation (7). Recent studies demonstrated that in cancer patients, sarcopenia may be viewed not just as a malnutritional alteration but also as a systemic inflammatory change (8, 9). Furthermore, cancer-induced changes in metabolism, inflammatory status, and hormonal regulation may in turn contribute to the development and progression of osteopenia and sarcopenia (9).

The intricate relationship between osteopenia, sarcopenia, and cancer outcomes is still unclear. Existing studies often focus on individual components—tumor characteristics, treatment modalities, and patient demographics—neglecting the combined impact of bone and muscle health on patient outcomes (10, 11). GI cancers often impair nutrient absorption, leading to deficiencies that contribute to bone loss and worse clinical outcomes. Osteopenia is linked to increased chemotherapy toxicity, poor surgical recovery, and higher recurrence rates, making it a valuable early predictor of prognosis (7, 12). Therefore, due to the strong association of osteopenia with malnutrition, sarcopenia, and cancer cachexia, all of which are prevalent in GI cancer patients, it is crucial to further assess its value as a potential prognostic marker in this type of cancer. This comprehensive systematic review and meta-analysis aim to evaluate the predictive value of osteopenia and osteosarcopenia as prognostic markers of survival and recurrence in patients with GI cancers. Our results may contribute to developing tailored interventions and improving the prognostic accuracy of GI cancer outcomes.

## Materials and methods

### Research questions

Is there an association between osteopenia and osteosarcopenia with outcomes such as overall survival (OS) and recurrence-free survival (RFS) among patients with gastrointestinal cancers?

### Objective

To evaluate the predictive value of osteopenia and osteosarcopenia as prognostic markers of survival and recurrence in patients with GI cancers.

### Inclusion and exclusion criteria (PECO)

#### Population

Cancer patients who underwent surgical resection following a pathologically diagnosed digestive tract cancer (gastric, colorectal, esophageal, liver, biliary tract, pancreatic, and gallbladder) were chosen as study participants.

#### Exposure

Preoperative osteopenia was the main exposure of interest. Osteopenia was defined using the BMD, in accordance with the individual studies (The individual author’s cut-offs for BMD were considered to categorize osteopenia). This study also included osteosarcopenia (coexistence of osteopenia and sarcopenia together). The definitions used for osteopenia and sarcopenia are elaborated in Table 1.

**Table 1:** Characteristics of included studies, *n* = 23.

Study	Country	Cancer type	Sample size	Study type	Measurement of osteopenia	Measurement of sarcopenia	Formula for osteopenia	Formula for sarcopenia	Age (median and range/ Mean (SD))	Inclusion criteria	Outcomes	Quality of study (NOS)
Takeda et al. (16)	Japan	Biliary tract cancer (BTC)	306	Retrospective	Non-contrast CT scan images at the level of the 11th thoracic vertebra were used	SMI analyzed at the level of L3 vertebra before surgery	cut-off of <135 HU	SMI < 42 cm2 /m2 for men and SMI < 38 cm2 /m2 for women	70 (64–76)	Patients diagnosed with unresectable or recurrent BTC	OS, DFS	8
Matsumoto et al. (17)	Japan	Extrahepatic biliary cancer (EHBC)	138	Retrospective	Non-contrast CT scan images at the level of the 11th thoracic vertebra were used	PMA at 3rd lumber vertebra	men = [308.82–2.49 × age]; women = [311.84–2.41 × age]	length of the major axes × length of the minor axes × *π*	71 (35–87)	Patients with EHBC underwent resection	OS, DFS	8
Miki et al. (18)	Japan	Intrahepatic Cholangiocarcinoma (IHCC)	71	Retrospective	Non-contrast CT scan images at the level of the 11th thoracic vertebra were used	CT scan images at the third lumbar spine (L3) level were used to measure the psoas muscle mass index (PMI)	cut-off of <160 HU	6.36 for men and 3.92 for women	68.3 ± 8.6	Adult patients who underwent hepatectomy for IHCC	Overall Survival (OS), Recurrence Free Survival (RFS)	8
Kato et al. (19)	Japan	Colorectal cancer (CRC)	1,086	Retrospective	Non-contrast CT scans at the level of the 11th thoracic vertebra were used to measure BMD	Not evaluated	308.82–2.49 × age in men and 311.84–2.41 × age in women	Not evaluated	69 (59–76)	Patients who underwent curative surgical resection of stage I to III CRC	OS, RFS	8
Yanagaki et al. (20)	Japan	Hepatocellular cancer (HCC)	227	Retrospective	Average pixel density within a circle in the mid-vertebral core at the bottom of the 11th thoracic vertebra (Th11) on preoperative computed tomography	Lengths of the major and minor axes of the psoas muscle at the caudal end of the third lumbar vertebra and calculated the area of the psoas muscle	308.82–2.49 × age in men and 311.84–2.41 × age in women	Skeletal muscle index (SMI) cut off of 11.0 cm2/m2 for men and 7.4 cm2/m2 for women	69 (62–74)	Patients with HCC who underwent primary hepatic resection	OS, RFS	8
Taniai et al. (21)	Japan	IHCC	41	Retrospective	BMD was measured in trabecular bone at the bottom of 11th thoracic vertebra (Th11) by calculating average pixel density within a circle	Psoas muscle mass area (PMA) below the sex-specific cutoffs level determined by a receiver-operating characteristics (ROC)	308.82–2.49 × age in men and 3.11.84–2.41 × age in female	major axis × the minor axis × π at the level of the 3^rd^ lumber vertebra	63 (55–68)	Patients with IHCC undergoing hepatic resection	OS, RFS	7
Abe et al. (22)	Japan	Pancreatic ductal adenocarcinoma (PDAC)	265	Retrospective	Average pixel density within an oval core at the level of the Th11 vertebra before surgery	SMI analyzed at the level of L3 vertebra before surgery	men: 308.82–2.49 × age (yr) and women: 311.84–2.41 × age (yr)	Cut-off preoperative SMI value of 47.1 and 36.6 for male and female patients	68.2 ± 8.3	Patients with no evidence of distant metastases and underwent surgical resection for PDAC	OS, RFS	9
Meister et al. (23)	Germany	HCC	176	Retrospective	At the level of 11th Thoracic vertebra	Not evaluated	cut-off of <175 HU	Not evaluated	79 (75, 84)	All patients who underwent partial hepatectomy for HCC	OS, RFS	7
Fukushima et al. (24)	Japan	Gastric cancer (GC)	224	Retrospective	Average pixel density within a circle of the mid-vertebral core at the bottom of the 11th thoracic vertebra (Th11) on preoperative plain CT	PMA at 3rd lumber vertebra	men = [308.82–2.49 × age]; women = [311.84–2.41 × age]	length of the major axes × length of the minor axes × π	73 (66–79)	Patients with GC underwent initial gastrectomy	OS, RFS	8
Takano et al. (25)	Japan	CRC	136	Retrospective	Average pixel density within a circle in the mid-vertebral core at the bottom of the Th11 on the preoperative plain CT image	Cross-sectional area (cm2) of skeletal muscle at the level of the third lumbar vertebra and normalizing it by the patient’s height (cm2/m2)	308.82–2.49 × age in men and 311.84–2.41 × age in women	SMI of ≤43.75 cm2/m2 for men and ≤ 41.10 cm2/m2 for women	72.6 (16.6) years	Stage I-III CRC aged 65–98 y who underwent curative resection.	OS, RFS	7
Watanabe et al. (26)	Japan	Perihilar Cholangiocarcinoma (PHCC)	256	Retrospective	Non-contrast CT scan images at the 11th thoracic (T11) vertebral level	Preoperative CT scan images at the level of the third lumbar (L3) vertebra	Cut-off of <160 HU	6.36 in males and 3.92 in females	70.3 ± 7.2	Patients who underwent resection of PHCC	OS	7
Cameron et al. (27)	United States	PDAC	152	Case control	Lumbar vertebral radiodensity (LVR)	An axial image at the level of the third lumbar (L3) vertebra	Not provided	Not provided	64.2 ± 12.6	Patients who underwent resection for histologically proven PDAC	OS	7
Kamada et al. (28)	Japan	CRC	230	Retrospective	Non-contrast CT images obtained at the 11th thoracic vertebra (Th11)	PMA at 3rd lumber vertebra	men = [308.82–2.49 × age]; women = [311.84–2.41 × age]	length of the major axes × length of the minor axes × π	67 (32–89 years)	Patients who underwent surgical resection for CRC	OS, RFS	7
Ikuta et al. (29)	Japan	Colorectal liver metastases (CRLM)	281	Retrospective	Non-contrast CT images obtained at the 11th thoracic vertebra	Not evaluated	BMD <141 HU	Not evaluated	66 (35–88 years)	Patients with CRLM underwent initial hepatic resection	OS, RFS	7
Furukawa et al. (30)	Japan	Colorectal liver metastases (CRLM)	118	Retrospective	Non-contrast CT images obtained at the 11th thoracic vertebra (Th11)	PMA at 3rd lumber vertebra	men = [308.82–2.49 × age]; women = [311.84–2.41 × age]	length of the major axes × length of the minor axes × π	Not provided	Patients with CRLM underwent initial hepatic resection	OS, RFS	8
Takahashi et al. (31)	Japan	Esophageal cancer (EC)	229	Retrospective	Average pixel density (HU) within a circle in the midvertebral core at the bottom of the 11th thoracic vertebra on preoperative CT	Cross-sectional area of the total skeletal muscle volume (cm2) at the bottom level of L3	Any	SMI < 41.1 cm2/m2 in females, and SMI < 43.0 cm2/m2 in males	65.3 ± 8.0	Patients with EC who underwent McKeown esophagectomy	OS, RFS	9
Tamura et al. (32)	Japan	EHCC	111	Retrospective	Non-contrast CT images obtained at the 11th thoracic vertebra (Th11)	The skeletal muscle area at the level of the third lumbar vertebra (L3) using transverse CT	308.82–2.49 × age in men and 311.84–2.41 × age in women	Not provided	Not provided	Patients who underwent PD	OS, RFS	7
Abe et al. (33)	Japan	PC	56	Retrospective	Non-contrast CT images obtained at the 11th thoracic vertebra (Th11)	The skeletal muscle area at the level of the third lumbar vertebra (L3) using transverse CT	Cut-off of <160 HU	The cut-off values were 38 cm2/m2 for women and 42 cm2/m2 for men	73 years	Patients who underwent pancreaticoduodenectomy (PD) or distal pancreatectomy (DP)	OS, RFS	9
Toshima et al. (34)	Japan	HCC	193	Retrospective	trabecular bone by calculating average pixel density within a circle in midvertebral core at the bottom of 11th thoracic vertebra	Cross-sectional areas (cm2) of skeletal muscles in L3 region	308.82–2.49 × age in men and 311.84–2.41 × age in women	126.9 x body surface area (BSA)–66.2 in men and 125.6 x BSA–81.1 in women	58 ± 6	Patients who underwent living donor liver transplantation	OS	8
Motomura et al. (35)	Japan	Pancreatic cancer (PC)	109	Retrospective	Non-contrast CT images at the Th11 level, using the entire vertebra body as the region of interest (ROI)	SMI analyzed at the level of L3 vertebra before surgery	Cut-off of <148 HU	Not provided	75 (49–90)	Patients who underwent resection for PC	OS, RFS	8
Sharshar et al. (36)	Japan	PC	181	Retrospective	BMD measurements were taken at the level of the 11th thoracic vertebra through calculation of the average pixel density within a circle	Psoas Muscle Index (PMI)	Males (137.5 HU) and females (128.8 HU)	Not provided	68 years (33–84)	Patients who underwent resection for PC	OS, RFS	7
Yao et al. (37)	Japan	EHBC	181	Case control	BMD measured by the CT attenuation value in the trabecular bone at the eleventh thoracic vertebral (Th11)	Psoas Muscle Index (PMI)	cut-off of <169 HU	Not provided	68 years (33–84)	Patients who underwent resection for EHBC	OS, RFS	7
Miyachi et al. (38)	Japan	HCC	465	Retrospective	BMD measured by the CT attenuation value in the trabecular bone at the eleventh thoracic vertebral (Th11)	Psoas Muscle Index (PMI)	cut-off of <160 HU	≤6.089	69 (62–75)	Patients underwent primary hepatectomy for HCC	OS, RFS	7

#### Outcome

The primary outcomes of interest were OS and RFS. OS was defined as the patient’s death between the date of resection and the last point of contact with the patient. RFS was calculated from the date of the tumor’s resection to the first recurrence at any site.

#### Study design

The review included all analytical designs, including cross-sectional, prospective, and retrospective studies.

### Exclusion criteria

Studies not reported in English, studies that were not retrievable, case reports, case series, and grey literature were excluded. The search was not restricted to a specific region or publication year.

Our literature search encompassed three databases: Medline, Google Scholar, and Science Direct, from inception until December 2023.

Primary and secondary data screenings were independently conducted by both authors. Any conflicts that arose between them were resolved through mutual consensus. The reporting of our review adhered to the Preferred Reporting Items for Systematic Reviews and Meta-Analyses (PRISMA) framework (13). During the primary screening, both authors screened titles and abstracts of the studies, removing any duplicates. In the subsequent secondary screening, full texts of the selected studies were reviewed using the inclusion criteria, and relevant information was extracted.

Both authors created and meticulously checked a data extraction template to ensure completeness and accuracy. Information such as author details, region, study design, inclusion criteria, type of cancer, sample size, definition of OS, SP, OSP, and the cut-offs used were extracted from individual studies and entered into the template.

The databases and PROSPERO were examined to ascertain the absence of prior systematic reviews on the same topic, confirming the novelty of our review (CRD42023493216).

### Search strategy

The following Medical subject heading (MeSH) terms were used: “Digestive tract cancer” OR “Digestive tract tumours” OR “Gastrointestinal neoplasms” AND “Osteopenia” OR “Low BMD” AND “Osteosarcopenia” AND “Survival” OR “Death” AND “Outcome” AND “Recurrence free survival” AND “Disease free survival” AND “Observational studies” OR “Cohort studies” OR “Prospective studies.” Reference list of included articles were screened for any potentially relevant studies. The detailed search strategy is provided as Supplementary material.

### Statistical analysis

All statistical analyses were performed using STATA 14.2. Binary outcomes (OS, DFS & RFS) were analyzed using the inverse variance method to combine effects across various studies, expressing outcomes as pooled hazards ratios (HR) with 95% confidence intervals (CIs). The Freeman-Tukey double arcsine transformation was applied to mitigate the potential influences of both large and small studies on pooled estimates. Diligent attempts were made to contact the authors for missing data. Results, presented as pooled effect sizes, were visually depicted through forest plots. Publication bias was assessed using funnel plots, and statistical tests were conducted using Egger’s test (14). Heterogeneity was assessed by *I*^2^ statistic and the Chi-square heterogeneity test. Heterogeneity levels were categorized as mild (*I*^2^ < 25%), moderate (*I*^2^ between 25 and 75%), and substantial (*I*^2^ > 75%). Due to expected heterogeneity in study definition and population, a random-effects model was used to account for the variation in effect sizes among the included studies. The between-study variance (*τ*^2^) was estimated using the Der Simonian and Laird technique, and the pooled hazard ratios (HRs) for survival outcomes were calculated using the inverse variance approach. *p* < 0.05 was statistically significant.

### Quality assessment of included studies

The Newcastle Ottawa Scale (NOS) (15) was used to evaluate study quality. This scale assesses studies based on outcomes, selection of study groups, and comparability, with a maximum score of nine for each study.

## Results

### Study selection

The initial search identified 1890 articles. After primary screening, 741 studies were removed as duplicates, and an additional 862 studies were removed at the stage of titles and abstracts evaluation. Of the remaining 287 articles, 54 free full-texts were retrieved for secondary screening, and 23 articles were ultimately selected for this systematic review and meta-analysis (16–38).

The reasons for exclusion were as follows: 18 studies reported on patients with other cancers, 15 did not define the exposure clearly, and 3 were not in English.

### Characteristics of the included studies

**Figure 1:**
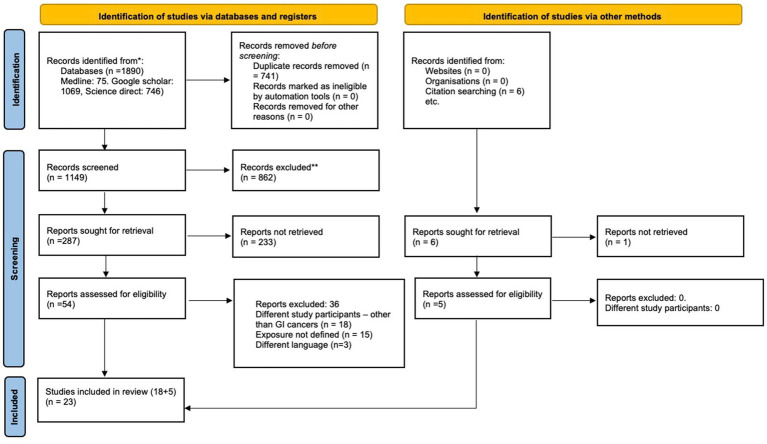
PRISMA 2020 flow diagram explaining the search flow.

The general characteristics of the included studies are outlined in Table 1. Of 23 studies, 21 were from Japan, and one study each was from Germany and the United States. Sample sizes of included studies ranged from 41 to 1,086. A majority (21/23) were retrospective. Figure 1 explains the study selection process. Twenty-one articles reported on the association between osteopenia and OS (18–38), 18 reported on the association between osteopenia and RFS (18–25, 28–33, 35–38). The association between OS and osteosarcopenia was reported by six studies (16, 17, 20–22, 25), RFS and osteosarcopenia were reported by five studies (17, 20–22, 25) and thus were pooled for the meta-analysis.

### Association between osteopenia (low BMD) with OS and DFS

**Figure 2:**
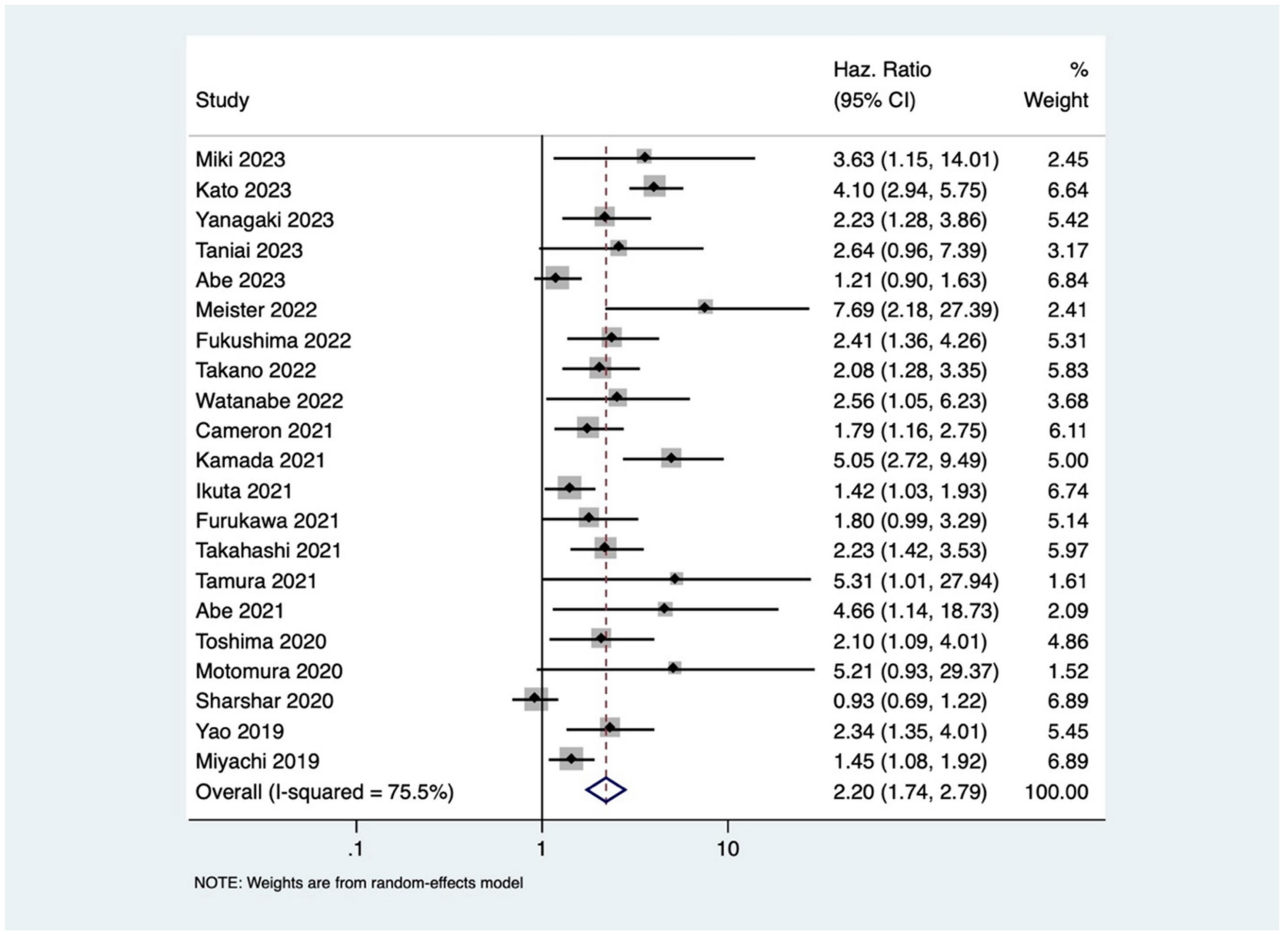
Forest plot of overall survival for osteopenia.

**Figure 3:**
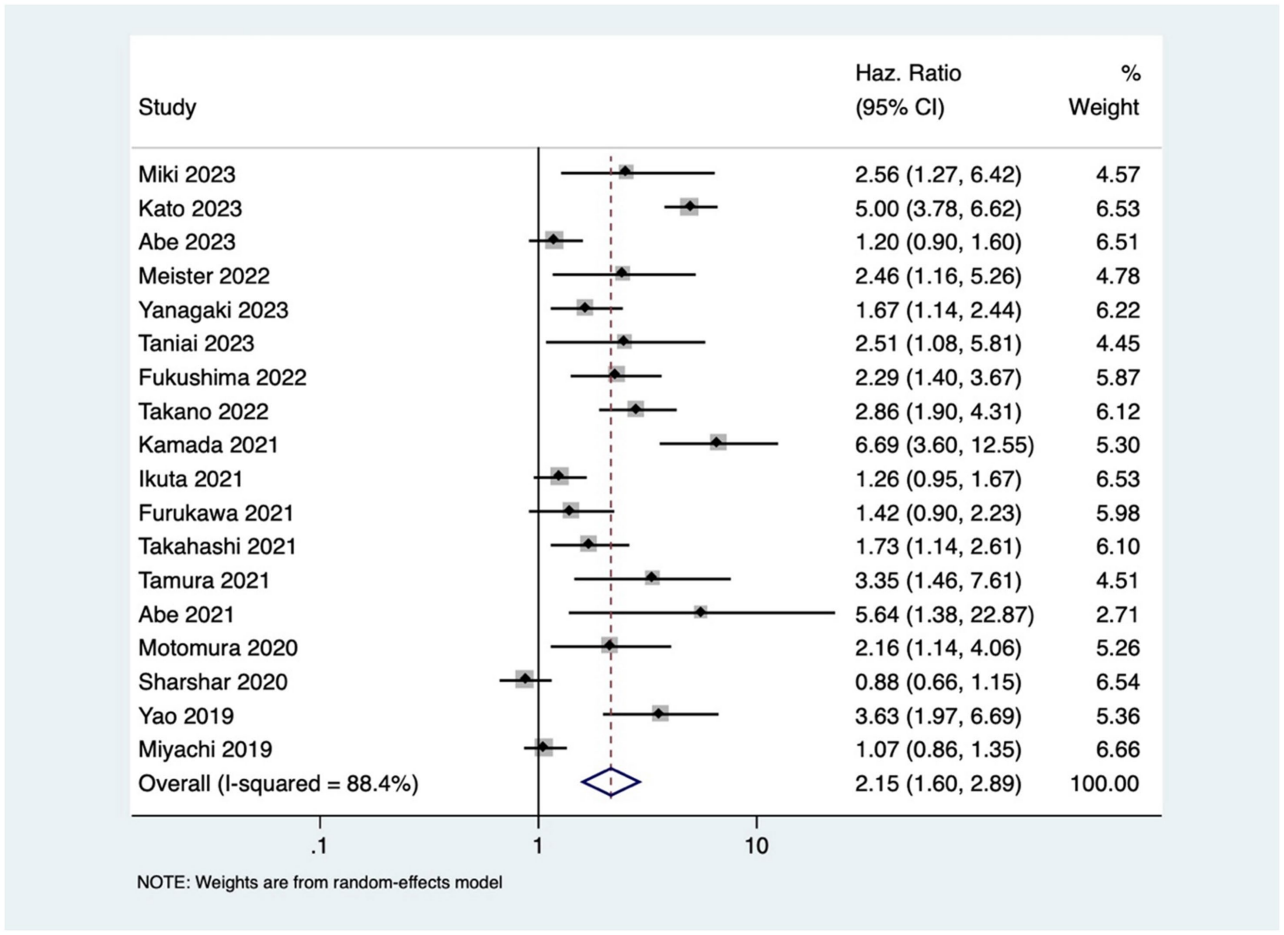
Forest plot of recurrence free survival for osteopenia.

Patients with osteopenia or low BMD had significantly poorer OS (pooled HR of 2.20, 95% CI: 1.74–2.79, with high heterogeneity *I*^2=^75.5, *p*-value <0.001) (Figure 2). The osteopenia was associated with lower RFS (pooled HR of 2.15, 95% CI: 1.60–2.89, with high heterogeneity *I*^2=^88.4, *p*-value <0.001) (Figure 3). Due to the high heterogeneity observed across the studies, subgroup analysis was done to investigate the reasons for clinical heterogeneity. The type of GI cancer, geographical region of included studies, and sample size showed a significant association between incidences of osteopenia and survival outcomes (except for the association between low BMD with OS and low BMD with RFS among pancreatic cancer patients) (Supplementary Figures 1–6).

### Association between osteosarcopenia with OS and DFS

**Figure 4:**
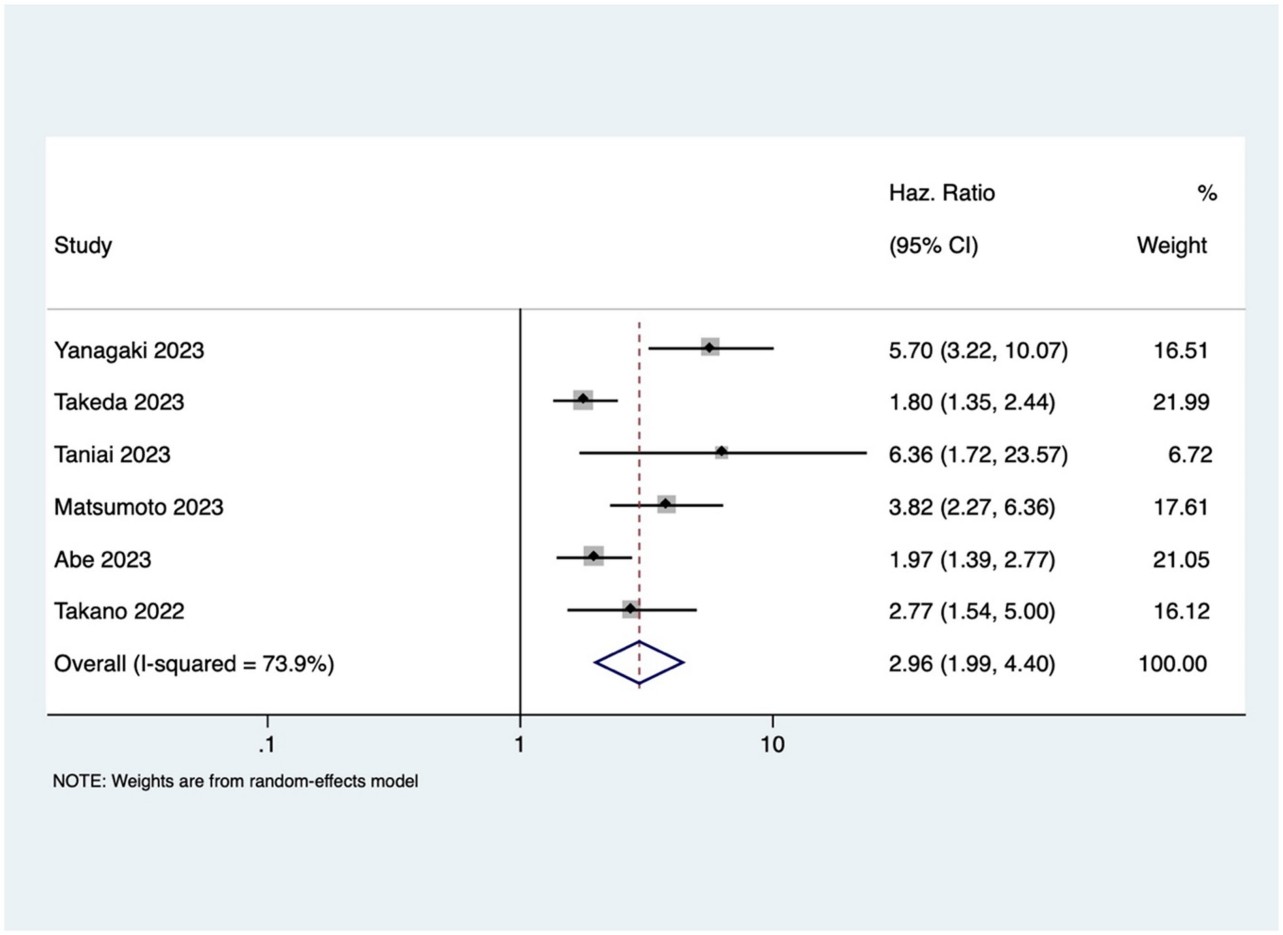
Forest plot of overall survival for osteosarcopenia.

**Figure 5:**
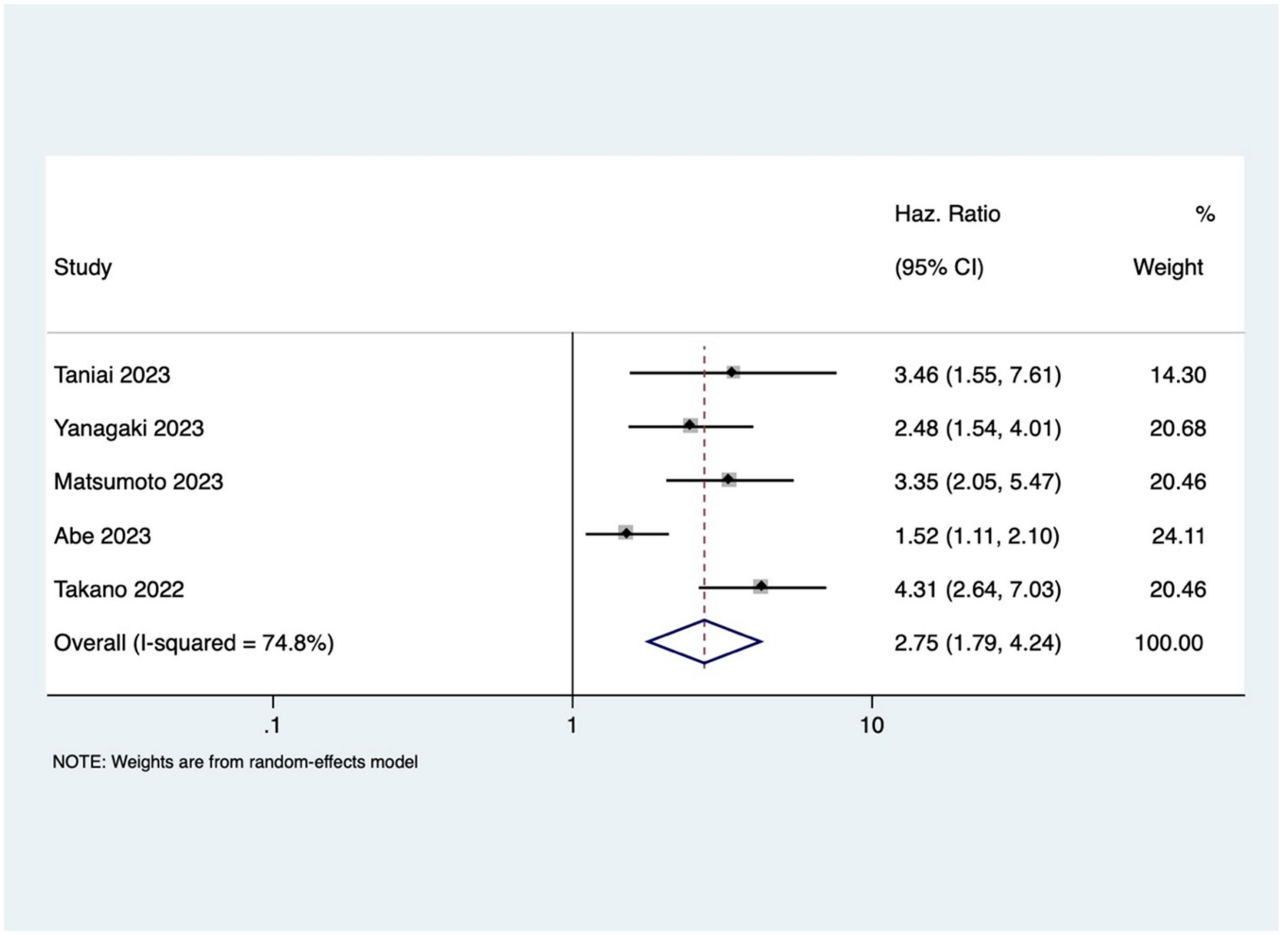
Forest plot of recurrence free survival for osteosarcopenia.

GI cancer patients with osteosarcopenia had three 3 times higher mortality risk compared to patients without osteosarcopenia (pooled HR 2.96, 95% CI: 1.99–4.40, with high heterogeneity *I*^2=^73.9, *p*-value <0.001) (Figure 4). Osteosarcopenia was a significant risk factor for poor RFS (pooled HR of 2.75, 95% CI: 1.79–4.24, with high heterogeneity *I*^2=^74.8, *p*-value <0.001) (Figure 5).

### Risk of bias

The asymmetric funnel plot showed evidence of publication bias for the association between osteopenia with OS and RFS (Supplementary Figures 7, 8).

Table 1 summarizes the risk of bias in the included studies, as assessed by NOS. Supplementary Figures 9, 10 show the effect of the risk of bias on the association between OS and RFS with osteopenia.

## Discussion

This meta-analysis showed that low BMD, osteopenia, and osteosarcopenia are potentially significant risk factors for poor OS and RFS among GI cancer patients. These findings highlight the need for preoperative assessment of GI cancer patients for timely interventions that may improve patient outcomes.

Together with genetics and ethnicity, BMD is a composite indicator reflecting exposure to multiple factors over the course of a patient’s life (19). BMD positively correlates with patient’s levels of estrogens, calcium and vitamin D intake, weight, and physical activity (39). Low BMD, therefore, is closely associated with factors that influence GI cancers either positively (calcium, vitamin D, oral contraceptives, physical activity) or negatively (age, BMI, smoking, alcohol) (40).

This study showed that GI cancer patients with osteopenia have 2-fold higher risk of death [pooled HR of 2.20, 95% CI: 1.74–2.79] and cancer recurrence [pooled HR of 2.15, 95% CI: 1.60–2.89]. These findings are comparable to the previous study done by Watanabe et al. that reported pooled HR of 2.02 and 1.96 for OS and RFS, respectively (41) The observed high heterogeneity in the association between osteopenia/osteosarcopenia and the outcomes might be attributed to variations in cancer types and stages, reflecting the heterogeneous nature of GI cancers. Despite the increased risk, the mechanism underlying osteopenia’s negative impact on prognosis is still unclear. One possible mechanism of this effect may be osteoclast stimulation brought on by cancer cachexia (severe, unintentional loss of weight, muscle mass, and strength due to chronic inflammation and metabolic dysfunction), resulting in bone loss (42). The compromised structural integrity of bones in patients with osteopenia may render them more susceptible to the skeletal complications of cancer (such as osteoporosis, fracture, and bone loss), contributing to the observed increased rates of mortality and cancer recurrence. Additionally, cytokines produced from cancer cells, such as PTHrP, interleukin (IL)-1, IL-6, and IL-8, create and activate osteoclasts through activating the RANK/RANKL receptors, and subsequently, NF-κB (43), which leads to muscle loss and sarcopenia (5, 44, 45). This study revealed that osteosarcopenia that encompasses both bone and muscle deficits was associated with 3-times higher mortality in GI cancer patients.

The interplay between chronic inflammation (increased IL-6 and TNF-*α* leading to osteoclast activation and muscle protein breakdown, increased NF-κB and RANKL expression), muscle-bone crosstalk dysregulation (myostatin overexpression, irisin and osteocyte dysfunction), metabolic dysfunction, and tumor microenvironment alterations (IGF-1 suppression and adipokines and endocrine dysfunction) underlies the association between osteopenia/osteosarcopenia and poor survival in GI cancers (44, 45).

The results of this study further corroborate other reports highlighting the compounded impact of this complex condition (46). While our findings were comparable with previous reports (16, 17, 22), the observed mortality rates associated with osteosarcopenia were slightly lower compared to other studies [HR >5] (20, 21). It is plausible that variations in study design, sample size, patient demographics, and follow-up period could cause this disparity. It’s possible that selection bias was more likely to affect earlier research with smaller sample numbers, which resulted in inflated hazard ratios. Inconsistencies between studies may have also been caused by differences in diagnostic thresholds, imaging modalities, and definitions of osteosarcopenia (47).

Additionally, this study showed that osteosarcopenia was associated with poorer RFS (pooled HR of 2.75; *p* < 0.001). This observation further emphasizes the need for a comprehensive assessment that includes both musculoskeletal aspects.

The subgroup analysis showed that osteopenia was associated with poor OS in patients with colorectal cancer (HR of 2.5) and lower RFS in patients with bile duct and colorectal cancer (HR of 3 and 2.75, respectively). These results are in agreement with the previous meta-analysis by Watanabe et al. that showed the highest mortality rates in patients with colorectal cancer in combination with osteopenia and a maximum risk for recurrence in patients with osteopenia and colorectal or bile duct cancer (41).

However, no association was detected in pancreatic cancer patients. This discrepancy may be due to the aggressive tumor biology and early metastatic spread of pancreatic cancer, which may overshadow the impact of osteopenia on survival. Additionally, treatment-related malabsorption (Whipple surgery leading to malabsorption, etc), cachexia, and vitamin D deficiencies might have confounded the relationship between survival and low bone mineral density. Variations in assessment methods, such as computer tomography (CT) vs. dual-energy X-ray absorptiometry (DXA) and heterogeneity in patient cohorts could also contribute to the inconsistency (48, 49).

It is also important to consider that cancer chemotherapies, including alkylating agents, FOLFIRI, antimetabolites, glucocorticoids, and platinum-derived cisplatin, cause direct dysregulation of bone turnover and nephrotoxicity, which expedite bone loss (46, 50). Additionally, low BMD-specific outcomes, especially frailty fractures, could significantly impair functional status and physical activity. This, in turn, could result in non-cancer mortality or non-adherence to cancer treatment, which triggers recurrence (51).

### Strengths and limitations

The main strengths of this review and meta-analysis are the inclusion of a substantial number of studies, rigorous screening processes, and comprehensive subgroup analyses that enhance the robustness of our findings.

However, this study has certain limitations. The high heterogeneity between the studies might impact the precision of our estimates.

One major limitation is the lack of standardized definitions for osteopenia and sarcopenia, which varied across the included studies. This might have introduced heterogeneity in the findings, affecting the comparability of results. Additionally, while DXA is considered a gold standard for diagnosing osteopenia, all studies included in this review diagnosed osteopenia using preoperative CT. Thus, over-reliance on CT-based measurements instead of DXA to assess BMD presents another challenge. Moreover, different studies used different threshold values for defining osteopenia. Most included studies were from Japan, thus limiting the generalisability of the findings. The predominance of Japanese studies in this meta-analysis raises concerns about the generalizability of our findings due to cultural, genetic, dietary, and healthcare system differences. Traditional Japanese diets, lower obesity rates, and distinct genetic factors influencing bone and muscle metabolism may affect the prevalence and impact of osteopenia differently than in Western populations. Finally, this study was unable to rule out the potential publication and language biases (since the review included only studies published in English).

## Conclusion and recommendations

In conclusion, this systematic review and meta-analysis show that osteopenia and osteosarcopenia are associated with significantly worse outcomes in patients with GI cancers. These results shed light on the intricate interplay between musculoskeletal health and outcomes in this population of patients. This study provides a robust foundation for integrating musculoskeletal assessments such as routine sarcopenia and osteopenia screening using tools like CT-based body composition analysis or DXA into the prognostic considerations for these cancers, and further strengthens the need of a holistic approach to GI cancer management that considers not only tumor characteristics but also patient’s bone and muscle health. Future research should also explore interventional strategies aimed at mitigating the negative impact of osteopenia and sarcopenia in GI cancer patients. Trials investigating the use of exercise therapies (resistance training and muscle mass training) nutritional supplementation, and pharmacological interventions (such as anti-resorptive agents like bisphosphonates or denosumab) among cancer patients with osteopenia and osteosarcopenia with standardized diagnostic criteria are necessary.

## Data Availability

Publicly available datasets were analyzed in this study. This data can be found here: Medline, Google Scholar, and Science Direct, from inception until December 2023.

## Supplementary material

SUPPLEMENTARY FIGURE 1 Forest plot of overall survival for osteopenia grouped by cancer type.

SUPPLEMENTARY FIGURE 2 Forest plot of overall survival for osteopenia grouped by country.

SUPPLEMENTARY FIGURE 3 Forest plot of overall survival for osteopenia grouped by sample size.

SUPPLEMENTARY FIGURE 4 Forest plot of recurrence free survival for osteopenia grouped by cancer type.

SUPPLEMENTARY FIGURE 5 Forest plot of recurrence free survival for osteopenia grouped by country.

SUPPLEMENTARY FIGURE 6 Forest plot of recurrence free survival for osteopenia grouped by sample size.

SUPPLEMENTARY FIGURE 7 Funnel plot of overall survival for osteopenia.

SUPPLEMENTARY FIGURE 8 Funnel plot of recurrence free survival for osteopenia.

SUPPLEMENTARY FIGURE 9 Funnel plot of overall survival for osteopenia by quality of included studies.

SUPPLEMENTARY FIGURE 10 Funnel plot of recurrence free survival for osteopenia by quality of included studies.

## References

[1] Siegel RL Miller KD Fuchs HE Jemal A. Cancer statistics, 2021. CA Cancer J Clin. (2021) 71:7–33. doi: 10.3322/caac.21654, PMID: 33433946

[2] Peery AF Crockett SD Murphy CC Lund JL Dellon ES Williams JL . Burden and cost of gastrointestinal, liver, and pancreatic diseases in the United States: update 2018. Gastroenterology. (2019) 156:254–272.e11. doi: 10.1053/j.gastro.2018.08.063, PMID: 30315778 PMC6689327

[3] Cruz-Jentoft AJ Bahat G Bauer J Boirie Y Bruyère O Cederholm T . Sarcopenia: revised European consensus on definition and diagnosis. Age Ageing. (2019) 48:601. doi: 10.1093/ageing/afz046, PMID: 31081853 PMC6593317

[4] Su H Ruan J Chen T Lin E Shi L. CT-assessed sarcopenia is a predictive factor for both long-term and short-term outcomes in gastrointestinal oncology patients: a systematic review and meta-analysis. Cancer Imaging. (2019) 19:82. doi: 10.1186/s40644-019-0270-0, PMID: 31796090 PMC6892174

[5] Verschueren S Gielen E O’Neill TW Pye SR Adams JE Ward KA . Sarcopenia and its relationship with bone mineral density in middle-aged and elderly European men. Osteoporos Int. (2013) 24:87–98. doi: 10.1007/s00198-012-2057-z 22776861

[6] Kirk B Zanker J Duque G. Osteosarcopenia: epidemiology, diagnosis, and treatment-facts and numbers. J Cachexia Sarcopenia Muscle. (2020) 11:609–18. doi: 10.1002/jcsm.12567, PMID: 32202056 PMC7296259

[7] Pin F Bonewald LF Bonetto A. Role of myokines and osteokines in cancer cachexia. Exp Biol Med. (2021) 246:2118–27. doi: 10.1177/15353702211009213, PMID: 33899538 PMC8524772

[8] Lavalle S Valerio MR Masiello E Gebbia V Scandurra G. Unveiling the intricate dance: how Cancer orchestrates muscle wasting and sarcopenia. In Vivo. (2024) 38:1520–9. doi: 10.21873/invivo.13602, PMID: 38936901 PMC11215601

[9] Looijaard SMLM Te Lintel Hekkert ML Wüst RCI Otten RHJ Meskers CGM Maier AB. Pathophysiological mechanisms explaining poor clinical outcome of older cancer patients with low skeletal muscle mass. Acta Physiol. (2021) 231:e13516. doi: 10.1111/apha.13516, PMID: 32478975 PMC7757176

[10] Clemente-Suárez VJ Redondo-Flórez L Rubio-Zarapuz A Martínez-Guardado I Navarro-Jiménez E Tornero-Aguilera JF. Nutritional and exercise interventions in Cancer-related Cachexia: an extensive narrative review. Int J Environ Res Public Health. (2022) 19:4604. doi: 10.3390/ijerph19084604, PMID: 35457471 PMC9025820

[11] Prado CM Purcell SA Laviano A. Nutrition interventions to treat low muscle mass in cancer. J Cachexia Sarcopenia Muscle. (2020) 11:366–80. doi: 10.1002/jcsm.12525, PMID: 31916411 PMC7113510

[12] Hirase Y Arigami T Matsushita D Shimonosono M Tsuruda Y Sasaki K . Prognostic significance of osteosarcopenia in patients with stage IV gastric cancer undergoing conversion surgery. Langenbeck's Arch Surg. (2024) 410:7. doi: 10.1007/s00423-024-03574-8, PMID: 39673019 PMC11645300

[13] Page MJ McKenzie JE Bossuyt PM Boutron I Hoffmann TC Mulrow CD . The PRISMA 2020 statement: an updated guideline for reporting systematic reviews. BMJ. (2021) 372:n71. doi: 10.1136/bmj.n71, PMID: 33782057 PMC8005924

[14] Egger M Davey Smith G Schneider M Minder C. Bias in meta-analysis detected by a simple, graphical test. BMJ. (1997) 315:629–34. doi: 10.1136/bmj.315.7109.629, PMID: 9310563 PMC2127453

[15] Lo CK-L Mertz D Loeb M. Newcastle-Ottawa scale: comparing reviewers’ to authors’ assessments. BMC Med Res Methodol. (2014) 14:45. doi: 10.1186/1471-2288-14-45, PMID: 24690082 PMC4021422

[16] Takeda T Okamoto T Sasaki T Hirai T Ishitsuka T Yamada M . The impact of osteosarcopenia in patients with unresectable or recurrent biliary tract cancer receiving palliative chemotherapy. Jpn J Clin Oncol. (2023) 53:1051–7. doi: 10.1093/jjco/hyad097, PMID: 37554052

[17] Matsumoto M Onda S Igarashi Y Hamura R Uwagawa T Furukawa K . Osteosarcopenia is a significant predictor of recurrence and the prognosis after resection for extrahepatic bile duct cancer. Surg Today. (2023) 54:407–18. doi: 10.1007/s00595-023-02747-0, PMID: 37700170

[18] Miki A Sakuma Y Watanabe J Endo K Sasanuma H Teratani T . Osteopenia is associated with shorter survival in patients with intrahepatic cholangiocarcinoma. Curr Oncol. (2023) 30:1860–8. doi: 10.3390/curroncol30020144, PMID: 36826105 PMC9955432

[19] Kato H Seishima R Mizuno S Matsui S Shigeta K Okabayashi K . The prognostic impact of preoperative osteopenia in patients with colorectal Cancer. Dis *Colon Rectum*. (2023) 66:e1225–33. doi: 10.1097/DCR.0000000000002961, PMID: 37699124

[20] Yanagaki M Haruki K Taniai T Igarashi Y Yasuda J Furukawa K . The significance of osteosarcopenia as a predictor of the long-term outcomes in hepatocellular carcinoma after hepatic resection. J Hepatobiliary Pancreat Sci. (2023) 30:453–61. doi: 10.1002/jhbp.1246, PMID: 36181339

[21] Taniai T Haruki K Yanagaki M Igarashi Y Furukawa K Onda S . Osteosarcopenia predicts poor prognosis for patients with intrahepatic cholangiocarcinoma after hepatic resection. Surg Today. (2023) 53:82–9. doi: 10.1007/s00595-022-02550-3, PMID: 35831486

[22] Abe T Nakata K Nakamura S Ideno N Ikenaga N Fujita N . Prognostic impact of preoperative Osteosarcopenia for patients with pancreatic ductal adenocarcinoma after curative resection. Ann Surg Oncol. (2023) 30:6673–9. doi: 10.1245/s10434-023-13936-z, PMID: 37466870

[23] Meister FA Verhoeven S Mantas A Liu W-J Jiang D Heij L . Osteopenia is associated with inferior survival in patients undergoing partial hepatectomy for hepatocellular carcinoma. Sci Rep. (2022) 12:18316. doi: 10.1038/s41598-022-21652-z, PMID: 36316524 PMC9622743

[24] Fukushima N Tsuboi K Nyumura Y Hoshino M Masuda T Suzuki T . Prognostic significance of preoperative osteopenia on outcomes after gastrectomy for gastric cancer. Ann Gastroenterol Surg. (2023) 7:255–64. doi: 10.1002/ags3.12635, PMID: 36998304 PMC10043770

[25] Takano Y Kodera K Tsukihara S Takahashi S Kobayashi Y Koyama M . Prognostic significance of osteosarcopenia in older adults with colorectal cancer. Ann Gastroenterol Surg. (2023) 7:637–44. doi: 10.1002/ags3.12663, PMID: 37416733 PMC10319614

[26] Watanabe J Miki A Sakuma Y Shimodaira K Aoki Y Meguro Y . Preoperative osteopenia is associated with significantly shorter survival in patients with Perihilar cholangiocarcinoma. Cancers. (2022) 14:2213. doi: 10.3390/cancers14092213, PMID: 35565342 PMC9103099

[27] Cameron ME Underwood PW Williams IE George TJ Judge SM Yarrow JF . Osteopenia is associated with wasting in pancreatic adenocarcinoma and predicts survival after surgery. Cancer Med. (2022) 11:50–60. doi: 10.1002/cam4.4416, PMID: 34791809 PMC8704155

[28] Kamada T Furukawa K Takahashi J Nakashima K Nakaseko Y Suzuki N . Prognostic significance of osteopenia in patients with colorectal cancer: a retrospective cohort study. Ann Gastroenterol Surg. (2021) 5:832–43. doi: 10.1002/ags3.12491, PMID: 34755015 PMC8560618

[29] Ikuta S Aihara T Nakajima T Kasai M Yamanaka N. Computed tomography-measured bone mineral density as a surrogate marker of survival after resection of colorectal liver metastases. Ann Transl Med. (2021) 9:21. doi: 10.21037/atm-20-3751, PMID: 33553314 PMC7859742

[30] Furukawa K Haruki K Taniai T Hamura R Shirai Y Yasuda J . Osteosarcopenia is a potential predictor for the prognosis of patients who underwent hepatic resection for colorectal liver metastases. Ann Gastroenterol Surg. (2021) 5:390–8. doi: 10.1002/ags3.12428, PMID: 34095730 PMC8164456

[31] Takahashi K Nishikawa K Furukawa K Tanishima Y Ishikawa Y Kurogochi T . Prognostic significance of preoperative osteopenia in patients undergoing Esophagectomy for esophageal Cancer. World J Surg. (2021) 45:3119–28. doi: 10.1007/s00268-021-06199-w, PMID: 34152448

[32] Tamura S Ashida R Sugiura T Okamura Y Ito T Yamamoto Y . The prognostic impact of skeletal muscle status and bone mineral density for resected distal cholangiocarcinoma. Clin Nutr. (2021) 40:3552–8. doi: 10.1016/j.clnu.2020.12.011, PMID: 33358552

[33] Abe K Furukawa K Okamoto T Matsumoto M Futagawa Y Haruki K . Impact of osteopenia on surgical and oncological outcomes in patients with pancreatic cancer. Int J Clin Oncol. (2021) 26:1929–37. doi: 10.1007/s10147-021-01986-w, PMID: 34232427

[34] Toshima T Yoshizumi T Kosai-Fujimoto Y Inokuchi S Yoshiya S Takeishi K . Prognostic impact of osteopenia in patients who underwent living donor liver transplantation for hepatocellular carcinoma. World J Surg. (2020) 44:258–67. doi: 10.1007/s00268-019-05206-5, PMID: 31624895

[35] Motomura T Uchiyama H Iguchi T Ninomiya M Yoshida R Honboh T . Impact of osteopenia on oncologic outcomes after curative resection for pancreatic Cancer. In Vivo. (2020) 34:3551–7. doi: 10.21873/invivo.12198, PMID: 33144467 PMC7811648

[36] Sharshar M Kaido T Shirai H Okumura S Yao S Miyachi Y . Impact of the preoperative bone mineral density on the outcomes after resection of pancreatic cancer. Surg Today. (2020) 50:757–66. doi: 10.1007/s00595-019-01954-y, PMID: 31925578

[37] Yao S Kaido T Okumura S Iwamura S Miyachi Y Shirai H . Bone mineral density correlates with survival after resection of extrahepatic biliary malignancies. Clin Nutr. (2019) 38:2770–7. doi: 10.1016/j.clnu.2018.12.004, PMID: 30595376

[38] Miyachi Y Kaido T Yao S Shirai H Kobayashi A Hamaguchi Y . Bone mineral density as a risk factor for patients undergoing surgery for hepatocellular carcinoma. World J Surg. (2019) 43:920–8. doi: 10.1007/s00268-018-4861-x, PMID: 30465085

[39] Dawson-Hughes B Harris SS. Calcium intake influences the association of protein intake with rates of bone loss in elderly men and women. Am J Clin Nutr. (2002) 75:773–9. doi: 10.1093/ajcn/75.4.773, PMID: 11916767

[40] Ganry O Lapôtre-Ledoux B Fardellone P Dubreuil A. Bone mass density, subsequent risk of colon cancer and survival in postmenopausal women. Eur J Epidemiol. (2008) 23:467–73. doi: 10.1007/s10654-008-9256-0, PMID: 18470627

[41] Watanabe J Saitsu A Miki A Kotani K Sata N. Prognostic value of preoperative low bone mineral density in patients with digestive cancers: a systematic review and meta-analysis. Arch Osteoporos. (2022) 17:33. doi: 10.1007/s11657-022-01060-6, PMID: 35149903 PMC8837550

[42] Sakuma K Hamada K Yamaguchi A Aoi W. Current nutritional and pharmacological approaches for attenuating sarcopenia. Cells. (2023) 12:2422. doi: 10.3390/cells12192422, PMID: 37830636 PMC10572610

[43] Ham SL Nasrollahi S Shah KN Soltisz A Paruchuri S Yun YH . Phytochemicals potently inhibit migration of metastatic breast cancer cells. Integr Biol. (2015) 7:792–800. doi: 10.1039/C5IB00121H, PMID: 26120051 PMC5474751

[44] Zain NM Seriramulu VP Chelliah KK. Bone mineral density and breast Cancer risk factors among premenopausal and postmenopausal women a systematic review. Asian Pac J Cancer Prev. (2016) 17:3229–34. doi: 10.14456/apjcp.2016.80/APJCP.2016.17.7.3229 27509955

[45] Stewart A Kumar V Torgerson DJ Fraser WD Gilbert FJ Reid DM. Axial BMD, change in BMD and bone turnover do not predict breast cancer incidence in early postmenopausal women. Osteoporos Int. (2005) 16:1627–32. doi: 10.1007/s00198-005-1886-4, PMID: 15782281

[46] Dolly A Lecomte T Bouché O Borg C Terrebonne E Douillard J-Y . Concurrent losses of skeletal muscle mass, adipose tissue and bone mineral density during bevacizumab / cytotoxic chemotherapy treatment for metastatic colorectal cancer. Clin Nutr. (2020) 39:3319–30. doi: 10.1016/j.clnu.2020.02.017, PMID: 32164981

[47] Xu F Xi H Liao M Zhang Y Ma H Wu M . Repurposed antipsychotic chlorpromazine inhibits colorectal cancer and pulmonary metastasis by inducing G2/M cell cycle arrest, apoptosis, and autophagy. Cancer Chemother Pharmacol. (2022) 89:331–46. doi: 10.1007/s00280-021-04386-z, PMID: 35067737

[48] Cao Y Efetov SK He M Fu Y Beeraka NM Zhang J . Updated clinical perspectives and challenges of chimeric antigen receptor-T cell therapy in colorectal Cancer and invasive breast Cancer. Arch Immunol Ther Exp. (2023) 71:19. doi: 10.1007/s00005-023-00684-x, PMID: 37566162

[49] Khalilieh S Iyer A Hammelef E Zohar N Gorgov E Yeo TP . Major pancreatic resection increases bone mineral density loss, osteoporosis, and fractures. Ann Surg. (2024). doi: 10.1097/SLA.0000000000006326. Epub ahead of print, PMID: 38775462

[50] Sturgeon KM Mathis KM Rogers CJ Schmitz KH Waning DL. Cancer- and chemotherapy-induced musculoskeletal degradation. JBMR Plus. (2019) 3:e10187. doi: 10.1002/jbm4.10187, PMID: 30918923 PMC6419610

[51] Cawthon PM Patel S Ewing SK Lui L Cauley JA Lyons JG . Bone loss at the hip and subsequent mortality in older men: The osteoporotic fractures in men (MrOS) study. JBMR Plus. (2017) 1:31–5. doi: 10.1002/jbm4.10006, PMID: 29124252 PMC5673261

