# Supplementary figures and images for: Predictive value of osteopenia as prognostic marker for survival and recurrence in patients with gastrointestinal cancers: a systematic review and meta-analysis
Source: Front Med (Lausanne). 2025 May 1;12:1527829. doi: 10.3389/fmed.2025.1527829 (PMC12078326; doi:10.3389/fmed.2025.1527829)

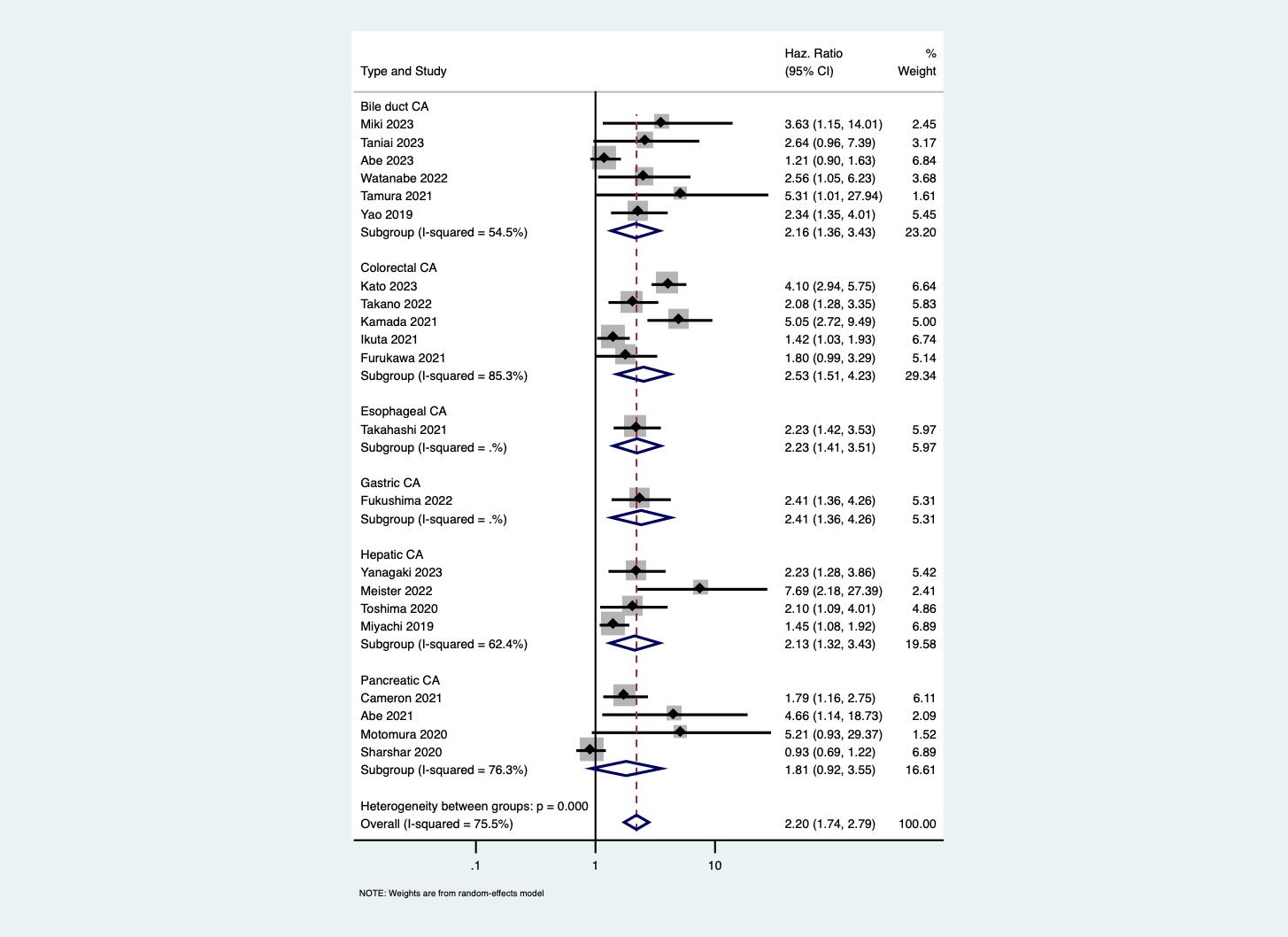

Supplement: SUPPLEMENTARY FIGURE 1 — Forest plot of overall survival for osteopenia grouped by cancer type. [file Image_1.JPEG]

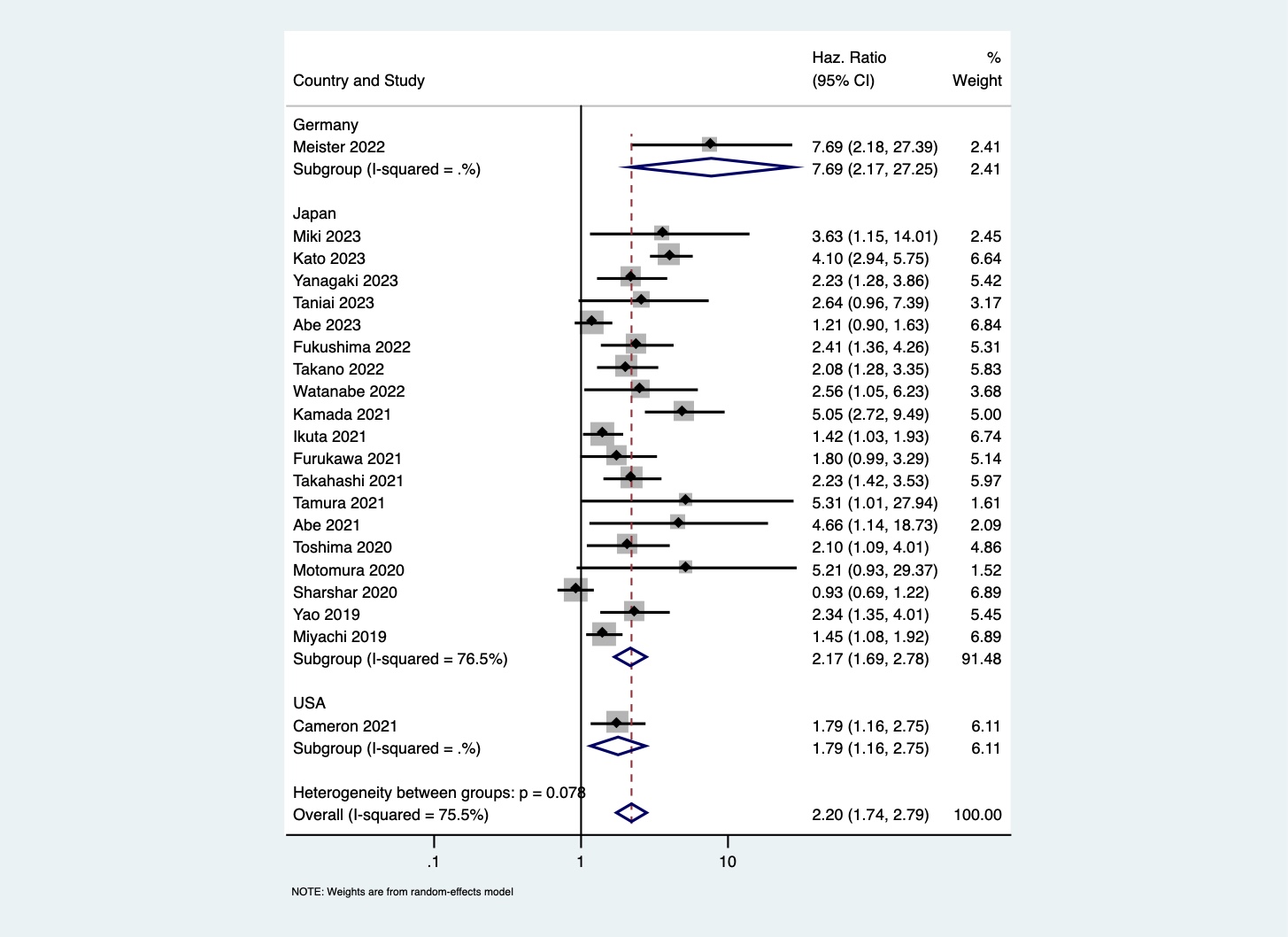

Supplement: SUPPLEMENTARY FIGURE 2 — Forest plot of overall survival for osteopenia grouped by country. [file Image_2.JPEG]

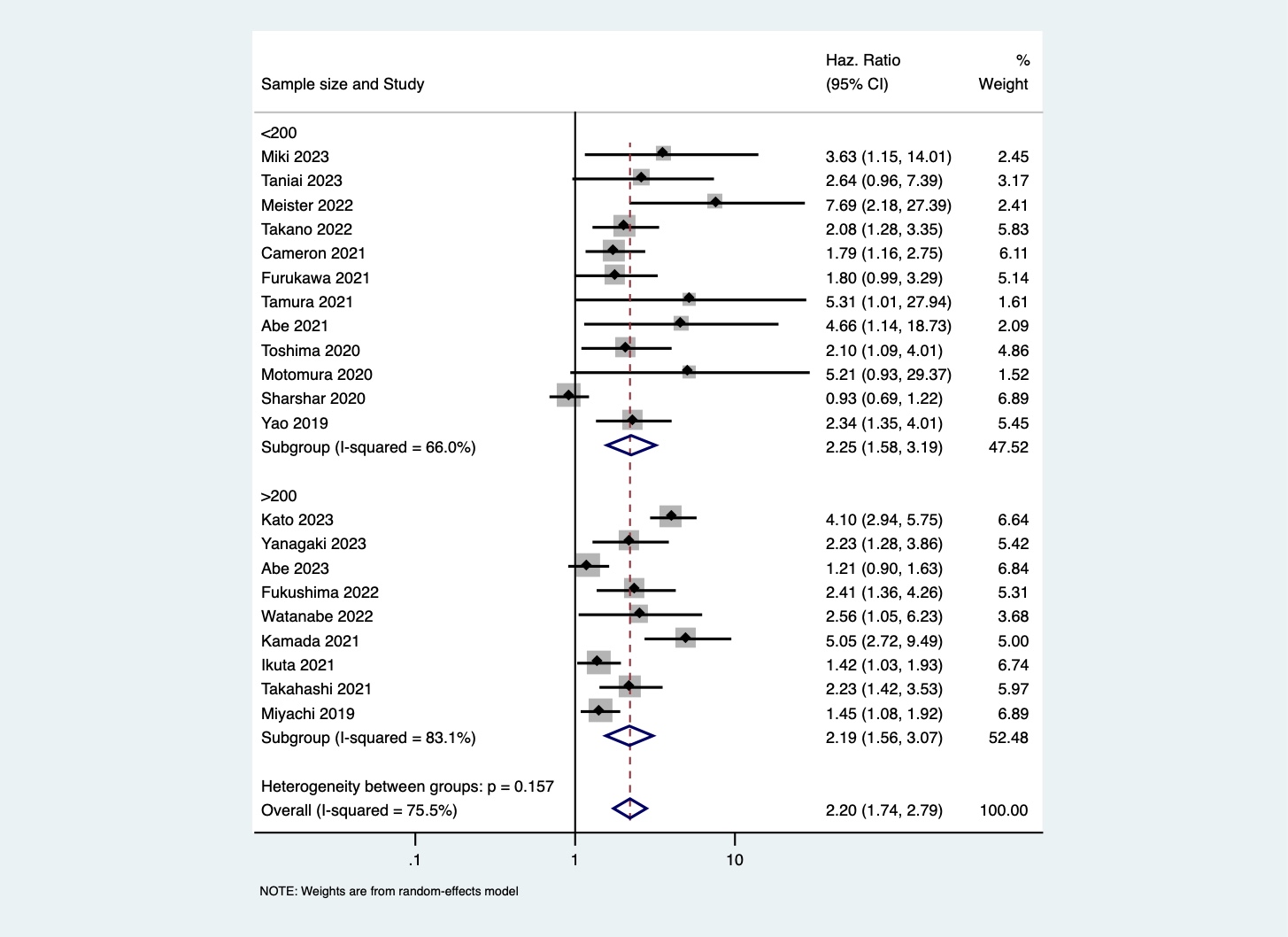

Supplement: SUPPLEMENTARY FIGURE 3 — Forest plot of overall survival for osteopenia grouped by sample size. [file Image_3.JPEG]

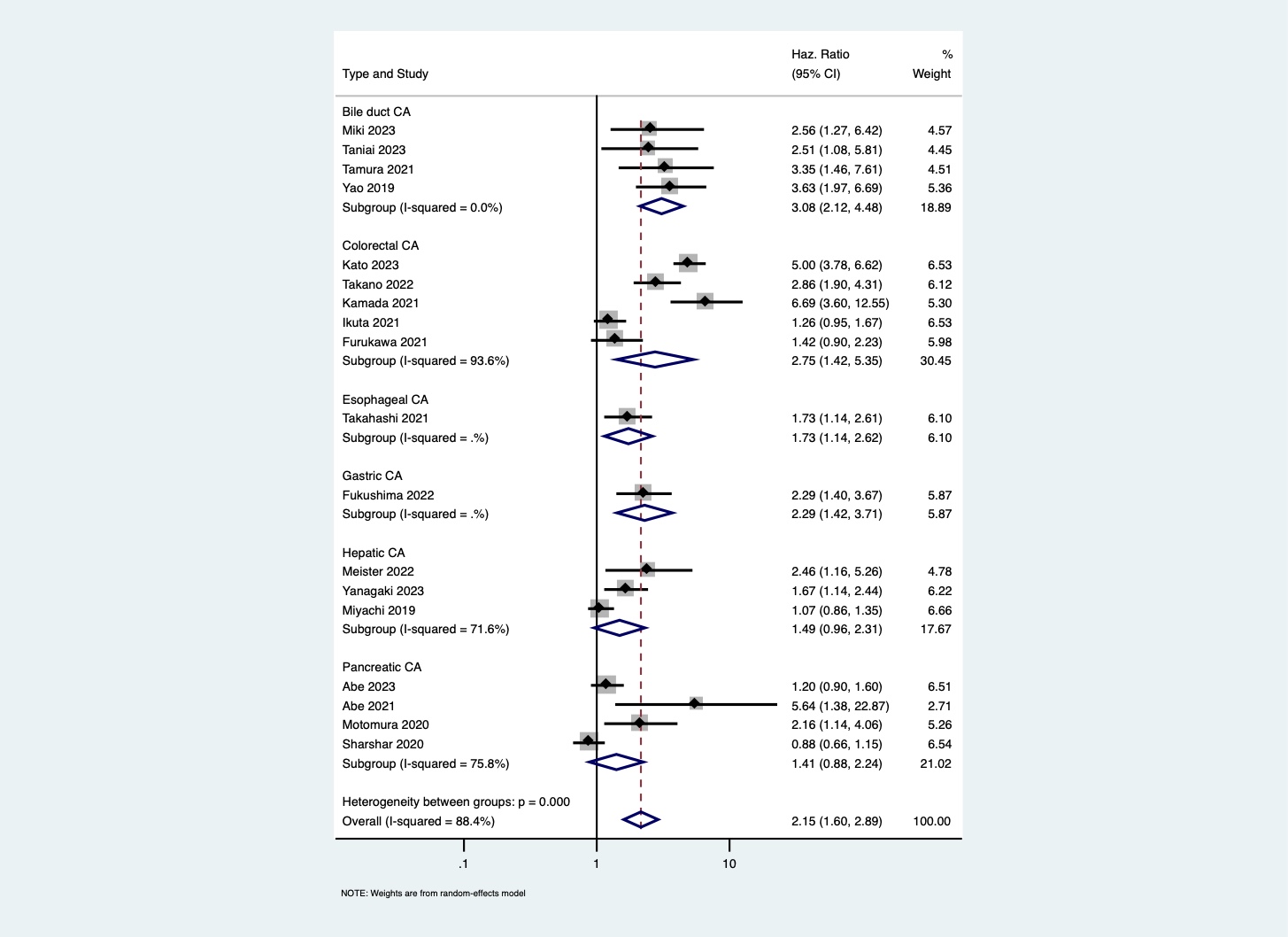

Supplement: SUPPLEMENTARY FIGURE 4 — Forest plot of recurrence free survival for osteopenia grouped by cancer type. [file Image_4.JPEG]

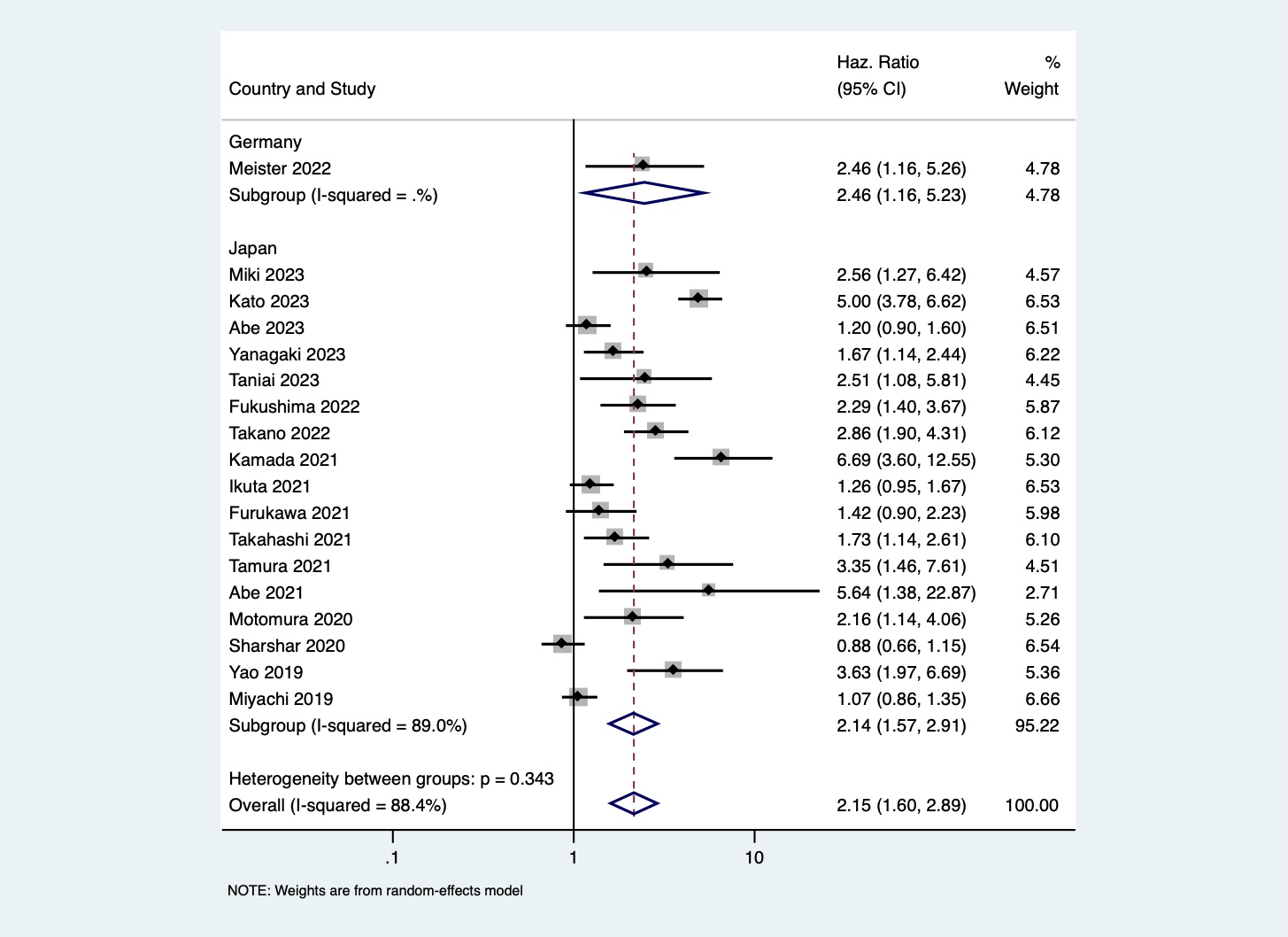

Supplement: SUPPLEMENTARY FIGURE 5 — Forest plot of recurrence free survival for osteopenia grouped by country. [file Image_5.JPEG]

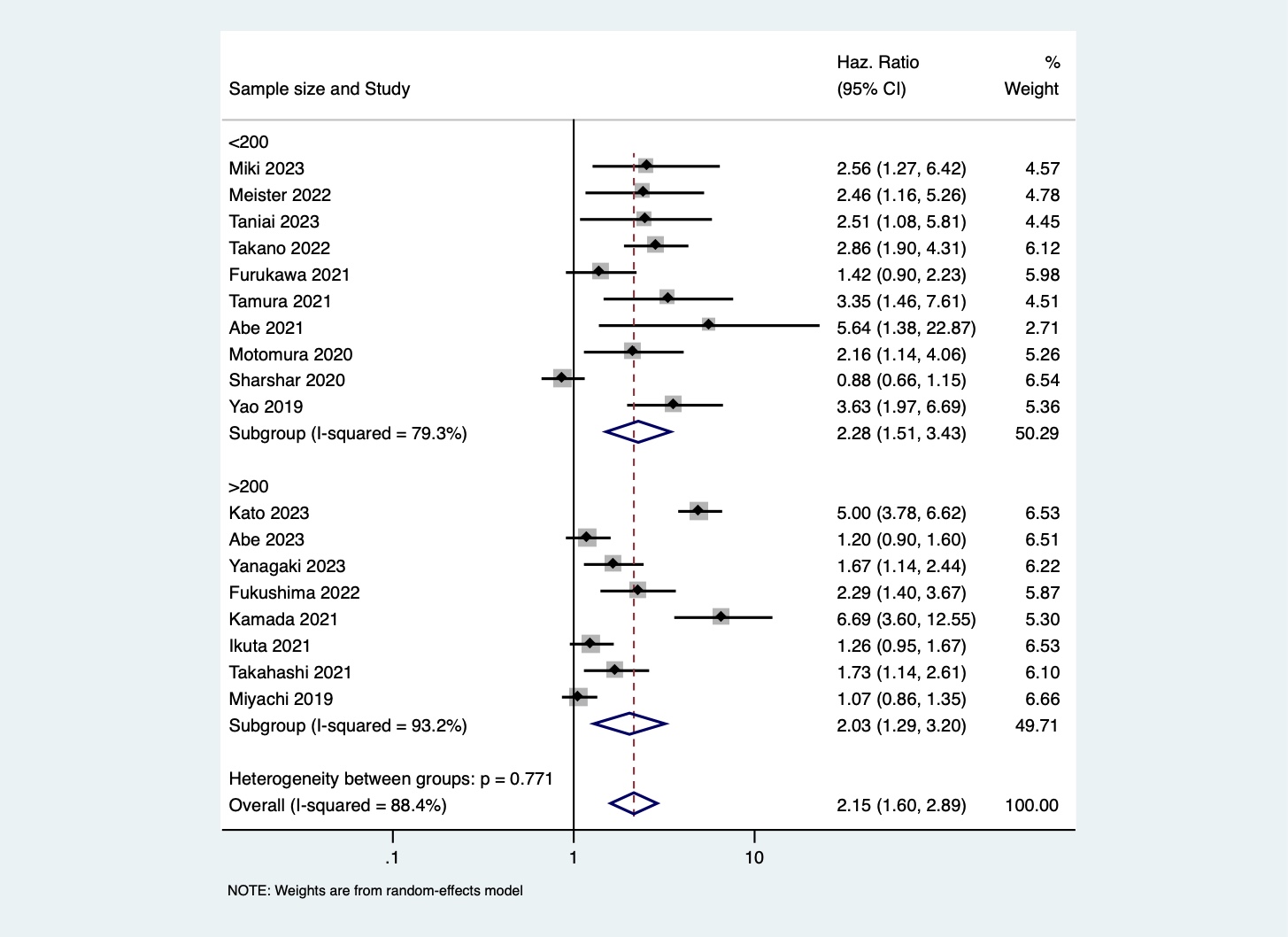

Supplement: SUPPLEMENTARY FIGURE 6 — Forest plot of recurrence free survival for osteopenia grouped by sample size. [file Image_6.JPEG]

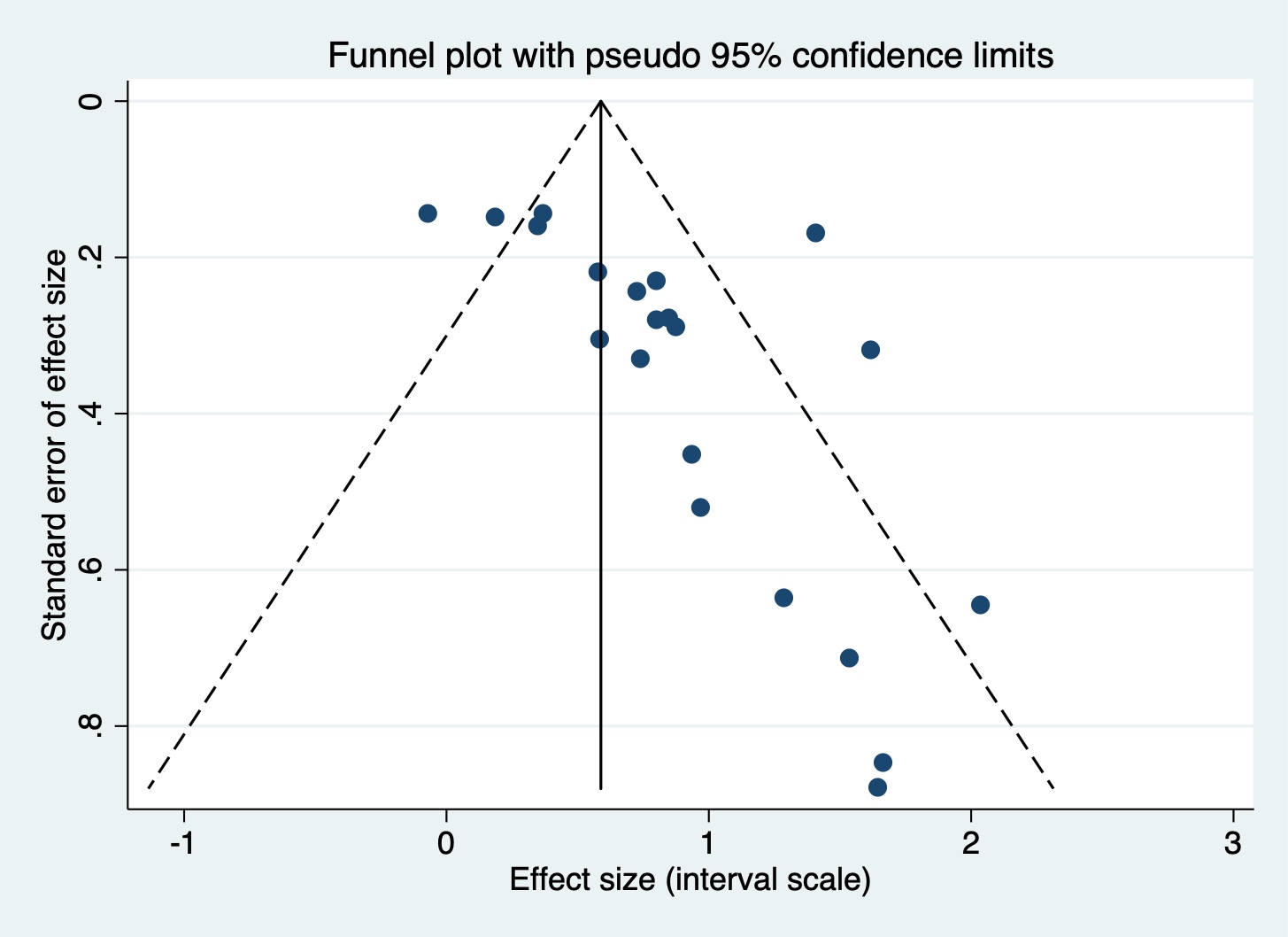

Supplement: SUPPLEMENTARY FIGURE 7 — Funnel plot of overall survival for osteopenia. [file Image_7.JPEG]

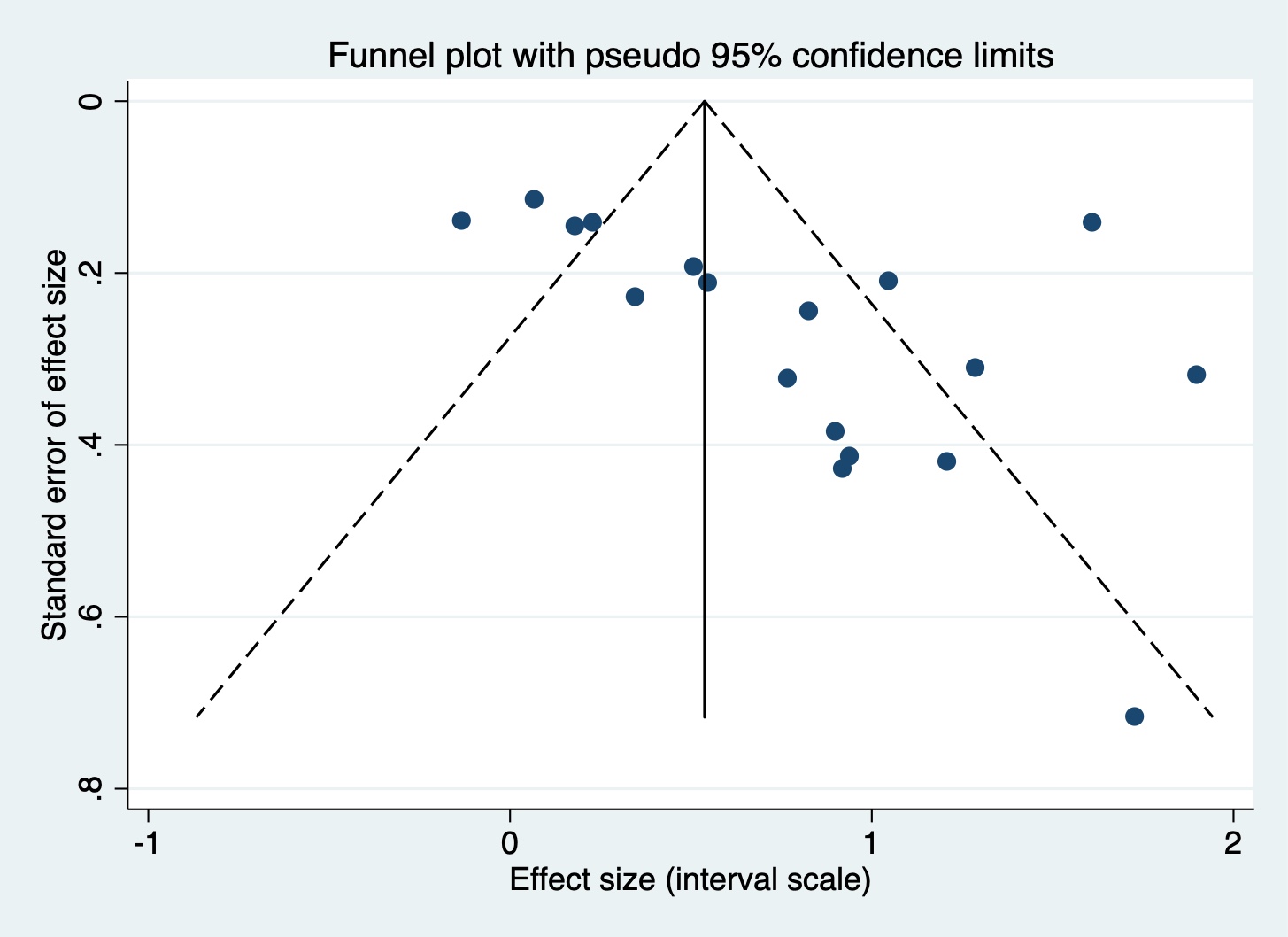

Supplement: SUPPLEMENTARY FIGURE 8 — Funnel plot of recurrence free survival for osteopenia. [file Image_8.JPEG]

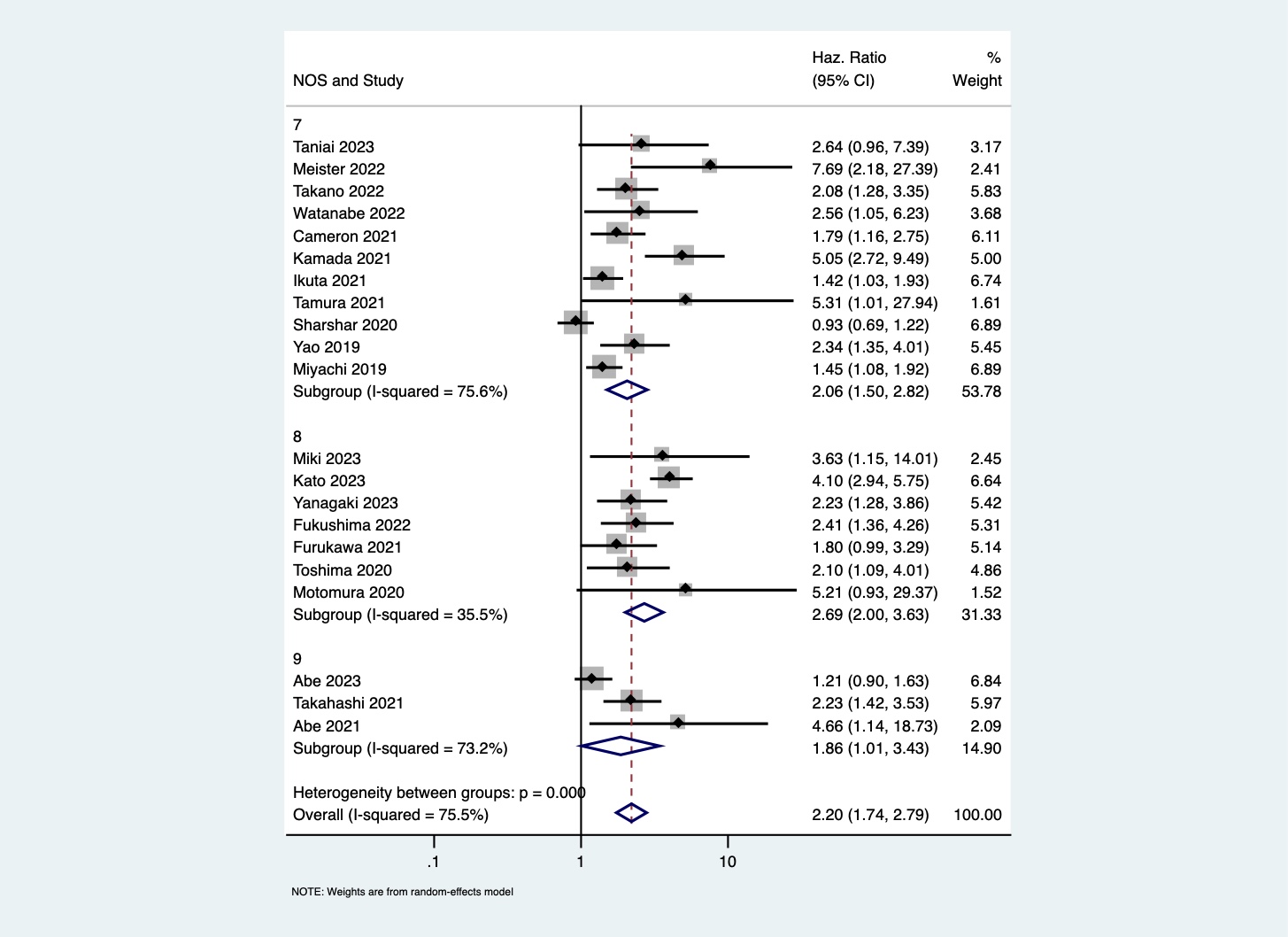

Supplement: SUPPLEMENTARY FIGURE 9 — Funnel plot of overall survival for osteopenia by quality of included studies. [file Image_9.JPEG]

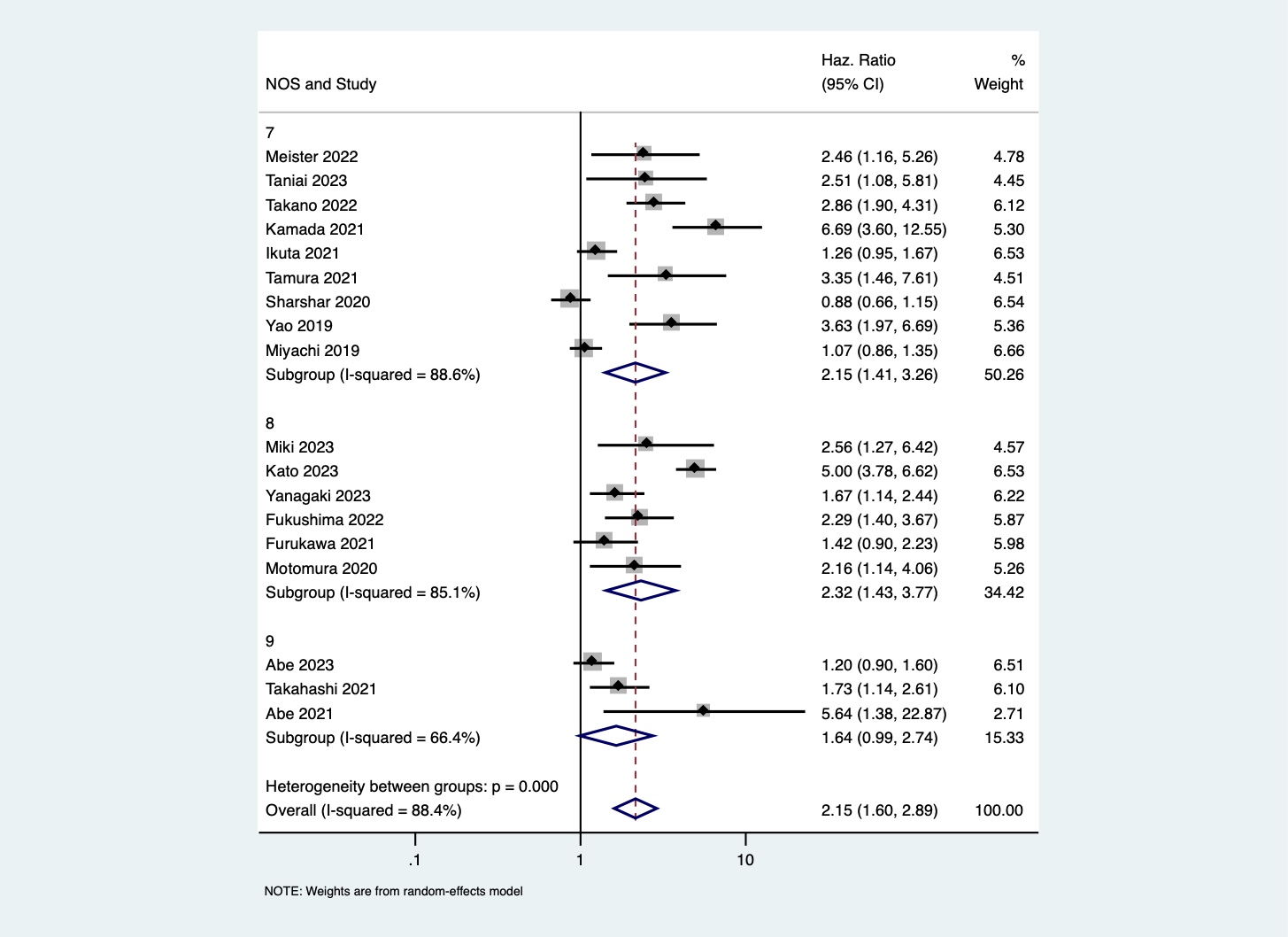

Supplement: SUPPLEMENTARY FIGURE 10 — Funnel plot of recurrence free survival for osteopenia by quality of included studies. [file Image_10.JPEG]
