# Supplementary material for: Predictive value of osteopenia as prognostic marker for survival and recurrence in patients with gastrointestinal cancers: a systematic review and meta-analysis
Source: Front Med (Lausanne). 2025 May 1;12:1527829. doi: 10.3389/fmed.2025.1527829 (PMC12078326; doi:10.3389/fmed.2025.1527829)
Supplement: Supplementary file 11 [file Table_1.DOCX]

Supplementary file 1: Search strategy

Pubmed

1. Search term: ((Digestive tract cancer) AND (Osteopenia)) AND (Survival)

Detail: ("gastrointestinal tract"[MeSH Terms] OR ("gastrointestinal"[All Fields] AND "tract"[All Fields]) OR "gastrointestinal tract"[All Fields] OR ("digestive"[All Fields] AND "tract"[All Fields]) OR "digestive tract"[All Fields]) AND ("cancer s"[All Fields] OR "cancerated"[All Fields] OR "canceration"[All Fields] OR "cancerization"[All Fields] OR "cancerized"[All Fields] OR "cancerous"[All Fields] OR "neoplasms"[MeSH Terms] OR "neoplasms"[All Fields] OR "cancer"[All Fields] OR "cancers"[All Fields]) AND ("bone diseases, metabolic"[MeSH Terms] OR ("bone"[All Fields] AND "diseases"[All Fields] AND "metabolic"[All Fields]) OR "metabolic bone diseases"[All Fields] OR "osteopenia"[All Fields] OR "osteopenias"[All Fields]) AND ("mortality"[MeSH Subheading] OR "mortality"[All Fields] OR "survival"[All Fields] OR "survival"[MeSH Terms] OR "survivability"[All Fields] OR "survivable"[All Fields] OR "survivals"[All Fields] OR "survive"[All Fields] OR "survived"[All Fields] OR "survives"[All Fields] OR "surviving"[All Fields])

Translations

*Digestive tract*: "gastrointestinal tract"[MeSH Terms] OR ("gastrointestinal"[All Fields] AND "tract"[All Fields]) OR "gastrointestinal tract"[All Fields] OR ("digestive"[All Fields] AND "tract"[All Fields]) OR "digestive tract"[All Fields]

*cancer*: "cancer's"[All Fields] OR "cancerated"[All Fields] OR "canceration"[All Fields] OR "cancerization"[All Fields] OR "cancerized"[All Fields] OR "cancerous"[All Fields] OR "neoplasms"[MeSH Terms] OR "neoplasms"[All Fields] OR "cancer"[All Fields] OR "cancers"[All Fields]

*Osteopenia*: "bone diseases, metabolic"[MeSH Terms] OR ("bone"[All Fields] AND "diseases"[All Fields] AND "metabolic"[All Fields]) OR "metabolic bone diseases"[All Fields] OR "osteopenia"[All Fields] OR "osteopenias"[All Fields]

*Survival*: "mortality"[Subheading] OR "mortality"[All Fields] OR "survival"[All Fields] OR "survival"[MeSH Terms] OR "survivability"[All Fields] OR "survivable"[All Fields] OR "survivals"[All Fields] OR "survive"[All Fields] OR "survived"[All Fields] OR "survives"[All Fields] OR "surviving"[All Fields]

1. Search term: ((Digestive tract cancer) AND (osteosarcopenia)) AND (Survival)

Detail: ("gastrointestinal tract"[MeSH Terms] OR ("gastrointestinal"[All Fields] AND "tract"[All Fields]) OR "gastrointestinal tract"[All Fields] OR ("digestive"[All Fields] AND "tract"[All Fields]) OR "digestive tract"[All Fields]) AND ("cancer s"[All Fields] OR "cancerated"[All Fields] OR "canceration"[All Fields] OR "cancerization"[All Fields] OR "cancerized"[All Fields] OR "cancerous"[All Fields] OR "neoplasms"[MeSH Terms] OR "neoplasms"[All Fields] OR "cancer"[All Fields] OR "cancers"[All Fields]) AND ("sarcopenia"[MeSH Terms] OR "sarcopenia"[All Fields] OR "sarcopenia s"[All Fields]) AND ("mortality"[MeSH Subheading] OR "mortality"[All Fields] OR "survival"[All Fields] OR "survival"[MeSH Terms] OR "survivability"[All Fields] OR "survivable"[All Fields] OR "survivals"[All Fields] OR "survive"[All Fields] OR "survived"[All Fields] OR "survives"[All Fields] OR "surviving"[All Fields])

Translations

*Digestive tract*: "gastrointestinal tract"[MeSH Terms] OR ("gastrointestinal"[All Fields] AND "tract"[All Fields]) OR "gastrointestinal tract"[All Fields] OR ("digestive"[All Fields] AND "tract"[All Fields]) OR "digestive tract"[All Fields]

*cancer*: "cancer's"[All Fields] OR "cancerated"[All Fields] OR "canceration"[All Fields] OR "cancerization"[All Fields] OR "cancerized"[All Fields] OR "cancerous"[All Fields] OR "neoplasms"[MeSH Terms] OR "neoplasms"[All Fields] OR "cancer"[All Fields] OR "cancers"[All Fields]

*Sarcopenia*: "sarcopenia"[MeSH Terms] OR "sarcopenia"[All Fields] OR "sarcopenia's"[All Fields]

*Survival*: "mortality"[Subheading] OR "mortality"[All Fields] OR "survival"[All Fields] OR "survival"[MeSH Terms] OR "survivability"[All Fields] OR "survivable"[All Fields] OR "survivals"[All Fields] OR "survive"[All Fields] OR "survived"[All Fields] OR "survives"[All Fields] OR "surviving"[All Fields]

1. Search term: ((Digestive tract cancer) AND (Osteopenia)) OR (osteosarcopenia) AND (Survival)

Details: ((("gastrointestinal tract"[MeSH Terms] OR ("gastrointestinal"[All Fields] AND "tract"[All Fields]) OR "gastrointestinal tract"[All Fields] OR ("digestive"[All Fields] AND "tract"[All Fields]) OR "digestive tract"[All Fields]) AND ("cancer s"[All Fields] OR "cancerated"[All Fields] OR "canceration"[All Fields] OR "cancerization"[All Fields] OR "cancerized"[All Fields] OR "cancerous"[All Fields] OR "neoplasms"[MeSH Terms] OR "neoplasms"[All Fields] OR "cancer"[All Fields] OR "cancers"[All Fields]) AND ("bone diseases, metabolic"[MeSH Terms] OR ("bone"[All Fields] AND "diseases"[All Fields] AND "metabolic"[All Fields]) OR "metabolic bone diseases"[All Fields] OR "osteopenia"[All Fields] OR "osteopenias"[All Fields])) OR "osteosarcopenia"[All Fields]) AND ("mortality"[MeSH Subheading] OR "mortality"[All Fields] OR "survival"[All Fields] OR "survival"[MeSH Terms] OR "survivability"[All Fields] OR "survivable"[All Fields] OR "survivals"[All Fields] OR "survive"[All Fields] OR "survived"[All Fields] OR "survives"[All Fields] OR "surviving"[All Fields])

Translations

*Digestive tract*: "gastrointestinal tract"[MeSH Terms] OR ("gastrointestinal"[All Fields] AND "tract"[All Fields]) OR "gastrointestinal tract"[All Fields] OR ("digestive"[All Fields] AND "tract"[All Fields]) OR "digestive tract"[All Fields]

*cancer*: "cancer's"[All Fields] OR "cancerated"[All Fields] OR "canceration"[All Fields] OR "cancerization"[All Fields] OR "cancerized"[All Fields] OR "cancerous"[All Fields] OR "neoplasms"[MeSH Terms] OR "neoplasms"[All Fields] OR "cancer"[All Fields] OR "cancers"[All Fields]

*Osteopenia*: "bone diseases, metabolic"[MeSH Terms] OR ("bone"[All Fields] AND "diseases"[All Fields] AND "metabolic"[All Fields]) OR "metabolic bone diseases"[All Fields] OR "osteopenia"[All Fields] OR "osteopenias"[All Fields]

*Survival*: "mortality"[Subheading] OR "mortality"[All Fields] OR "survival"[All Fields] OR "survival"[MeSH Terms] OR "survivability"[All Fields] OR "survivable"[All Fields] OR "survivals"[All Fields] OR "survive"[All Fields] OR "survived"[All Fields] OR "survives"[All Fields] OR "surviving"[All Fields]

Science direct

Search term: (((Digestive tract cancer) AND (Osteopenia)) AND (osteosarcopenia)) AND (Survival)

Google scholar

Keywords: Digestive tract cancers; Gastrointestinal neoplasms; Osteopenia; Low BMD; Osteosarcopenia; Overall survival; Disease free survival; Recurrence free survival; Cohort studies; Case-control studies; Cross-sectional studies; Observational studies
